# Deliberate Facial Mimicry As a Skill That Predicts Emotion Recognition Performance

**DOI:** 10.1007/s42761-026-00366-9

**Published:** 2026-04-16

**Authors:** Liron Amihai, Shachar Maer, Daniel Toledano, Galit Yovel, Yaara Yeshurun

**Affiliations:** 1 School of Psychological Sciences, Tel Aviv University, Tel Aviv, Israel; 2 Sagol School of Neuroscience, Tel Aviv University, Tel Aviv, Israel

**Keywords:** Facial mimicry, Emotion recognition, Embodied simulation, Individual differences, Drift-diffusion

## Abstract

How do we recognize emotions in others’ faces? Embodied-simulation theory suggests we mimic observed expressions to enhance our understanding. However, empirical evidence for this online mimicry account remains mixed. Prior research typically measured facial mimicry and emotion recognition concurrently. Here, we test a complementary embodied-simulation account by measuring deliberate facial mimicry quality as an independent skill. We test whether individual differences in this skill - potentially reflecting richer sensorimotor representations - predict emotion recognition performance. In Study 1 (*N* = 34), participants completed three facial-emotion-recognition tasks, then performed an instructed-mimicry task. We developed and validated a deep-learning-based algorithm to quantify mimicry accuracy and timing, applied diffusion modeling to capture recognition efficiency, and linked mimicry skill to emotion recognition performance. Study 2 (*N* = 50) was a preregistered replication. Results showed that accurate mimickers recognized emotions more precisely and efficiently. Additionally, longer mimicry latencies predicted higher recognition accuracy. Our findings link facial mimicry quality and emotion recognition performance, highlighting mimicry as a measurable skill that predicts social-cognitive ability.

**Supplementary Information:**

The online version contains supplementary material available at 10.1007/s42761-026-00366-9.

Our faces are our most powerful social instruments - constantly broadcasting, receiving, and mirroring the emotions of those around us (Darwin, 1872; Fridlund, 2014). We spontaneously and sometimes deliberately recreate the facial expressions we witness in others (Dimberg et al., 2000; Niedenthal et al., 2010). In this study we seek to reveal whether one’s ability to recreate others’ facial expressions with high precision correlates with one’s capacity to understand emotional expressions. Previous studies measured facial mimicry and facial expression recognition concurrently. Here, we adopt an individual differences approach and investigate whether the quality of instructed facial mimicry is a trait that predicts facial-emotion recognition.

Recognition of facial expressions is a fundamental aspect of human social interaction, serving as a critical component of non-verbal communication (Van Kleef & Côté, 2022; Adolphs, 2002). Embodied simulation theory proposes that observers understand others’ actions, intentions, and mental states by simulating or “mirroring” these observed behaviors through their own sensorimotor system (Gallese, 2005). In that sense, facial mimicry, the time-locked replication of observed emotional expressions (Dimberg, 1982; Hess & Fischer, 2024), may serve as embodied simulation mechanism (Goldman & Sripada, 2005). As a precursor to emotional contagion, facial mimicry also enables the interpersonal transfer of affect (Prochazkova & Kret, 2017; Hatfield et al., 2014; but see Olszanowski et al., 2020).

The role of facial mimicry in emotion recognition remains a subject of ongoing debate in literature. It was suggested that through sensorimotor processes, observers partially recreate others’ emotional states, enabling facial feedback that allows individuals to experience and better understand observed emotions during recognition tasks (Niedenthal, 2007; Niedenthal et al., 2017; Stel & Van Knippenberg, 2008). Supporting evidence shows that congruent facial muscle activity facilitates recognition (Folz et al., 2023; Künecke et al., 2014), and restricting mimicry slows expression change detection (Niedenthal et al., 2001) and impairs distinguishing authentic from fake expressions (Rychlowska et al., 2014). However, contrary evidence shows facial movement restriction doesn’t always impair recognition (Kosonogov et al., 2015), instructed mimicry may sometimes reduce accuracy (Kulesza et al., 2015; Drimalla et al., 2021), and a meta-analysis found non-significant overall effects (Holland et al., 2021), highlighting ongoing ambiguity about mimicry’s contribution to emotion recognition.

Clinical evidence from individuals with facial movement impairments also reveals mixed findings. Supporting a mimicry-recognition link, individuals with Parkinson’s disease show reduced happiness recognition, and those with Locked-in Syndrome show impaired negative emotion recognition (Argaud et al., 2016; Pistoia et al., 2010). In autism spectrum disorders, explicit mimicry instructions improve emotion recognition despite impaired spontaneous mimicry (Lewis & Dunn, 2017; Yoshimura et al., 2015), and individuals high in alexithymia exhibit reduced mimicry and poorer expression decoding (Franz et al., 2021; Nordmann et al., 2021; Parker et al., 1993; Rosenberg et al., 2020). However, Moebius syndrome studies show mixed results—some find no recognition deficits (Bogart & Matsumoto, 2010; Vannuscorps et al., 2020), others report specific impairments for fear and happiness (Japee et al., 2023), and others propose that mere ability to produce facial expressions may determine recognition capacity (Japee et al., 2024). Additionally, in schizophrenia, mimicry remains intact but decouples from recognition accuracy (Torregrossa et al., 2019), suggesting mimicry alone may be insufficient for optimal recognition.

Given the mixed evidence surrounding the role of facial mimicry in emotion recognition, we address this question through the lens of individual differences rather than experimental manipulation. Embodied-simulation accounts propose that observers recognize facial expressions by simulating the motor plans, somatosensory states, and affective responses involved in producing those expressions themselves, drawing on stored multimodal emotion representations shaped by prior expressive experience (Niedenthal et al., 2010; Wood et al., 2016; Ross & Atkinson, 2020). Importantly, these models acknowledge that mimicry is not always necessary - perceptual input alone may suffice for clear, prototypical expressions - but posit that it becomes especially informative when expressions are subtle, ambiguous, or context dependent (Niedenthal et al., 2010). Prior empirical work has primarily targeted the online simulation component of this framework by measuring or manipulating spontaneous facial mimicry while participants simultaneously categorize emotional expressions (e.g., Argaud et al., 2016; Borgomaneri et al., 2020; Ponari et al.,^2012^; Stel & Van Knippenberg, 2008).

Here, we focus on a complementary aspect: the richness and precision of the underlying sensorimotor representations themselves. Although spontaneous mimicry is most commonly linked to online embodied simulation, deliberate mimicry quality may likewise reflect the fidelity of an individual’s learned visuomotor mappings for emotional expressions (Cook et al., 2013; Japee, 2024) - such that the same representational substrate supporting accurate production also supports perceptual processing (Wood et al., 2016). We therefore propose that measuring deliberate facial mimicry quality as a standalone skill offers a behavioral window into these embodied emotion representations and, in turn, should predict individual differences in emotion recognition ability across tasks.

To test this, we introduce a novel computational method for quantifying mimicry accuracy and temporal dynamics from video-to-video comparison of dynamic expressions. We then test whether individual differences in these mimicry skills predict emotion recognition performance across separate, independent tasks. We tested this in two studies using four tasks: a naturalistic deliberate facial mimicry task and three expression recognition tasks that capture different facets of emotion recognition. Study 1 served as an exploratory investigation with two primary goals: (1) developing and validating a novel computational method for measuring deliberate facial mimicry accuracy, and (2) testing whether individual differences in mimicry skill predict emotion recognition performance. Study 2 was then designed as a preregistered confirmatory replication.

## Study 1

### Method

#### Participants

Thirty-four participants (22 female, 12 male), aged 18 to 35 years (*M *= 24.44, *SD* = 4.03), took part in the experiment. The study was approved by the university’s ethics committee. All participants provided written informed consent to participate in the study and received monetary compensation or course credit for their time. We determined our sample size by examining related studies (Cook et al., 2013, *N* = 20; Williams et al., 2013, *N* = 24; Perugia et al., 2021, *N* = 45) and selecting a sample size consistent with this literature (see Study 2 power analysis that is based on study 1’s results).

#### Procedure

We assessed participants’ facial mimicry capacity with the Deliberate Mimicry Task, and their emotion recognition capacity with three tasks: Expression-Name Congruency, Stop-and-Identify, and Film Task. These tasks capture different facets of emotion recognition (perceptual matching, dynamic detection, and fine-grained discrimination), providing a broad assessment of facial expression proficiency with sufficient variability for drift-diffusion analysis. Each task is described below. During all tasks, participants were seated in a quiet room, approximately 70 cm from a 23.8” monitor. The experimental tasks were presented in full-screen mode using either PsychoPy (Peirce, 2007) or jsPsych (De Leeuw, 2015), depending on the task. Tasks were completed in a fixed order: Expression-Name Congruency, Stop-and-Identify, Film Task, and finally the Deliberate Mimicry Task.

#### Facial Expressions Recognition (FER) Tasks

**Fig. 1:**
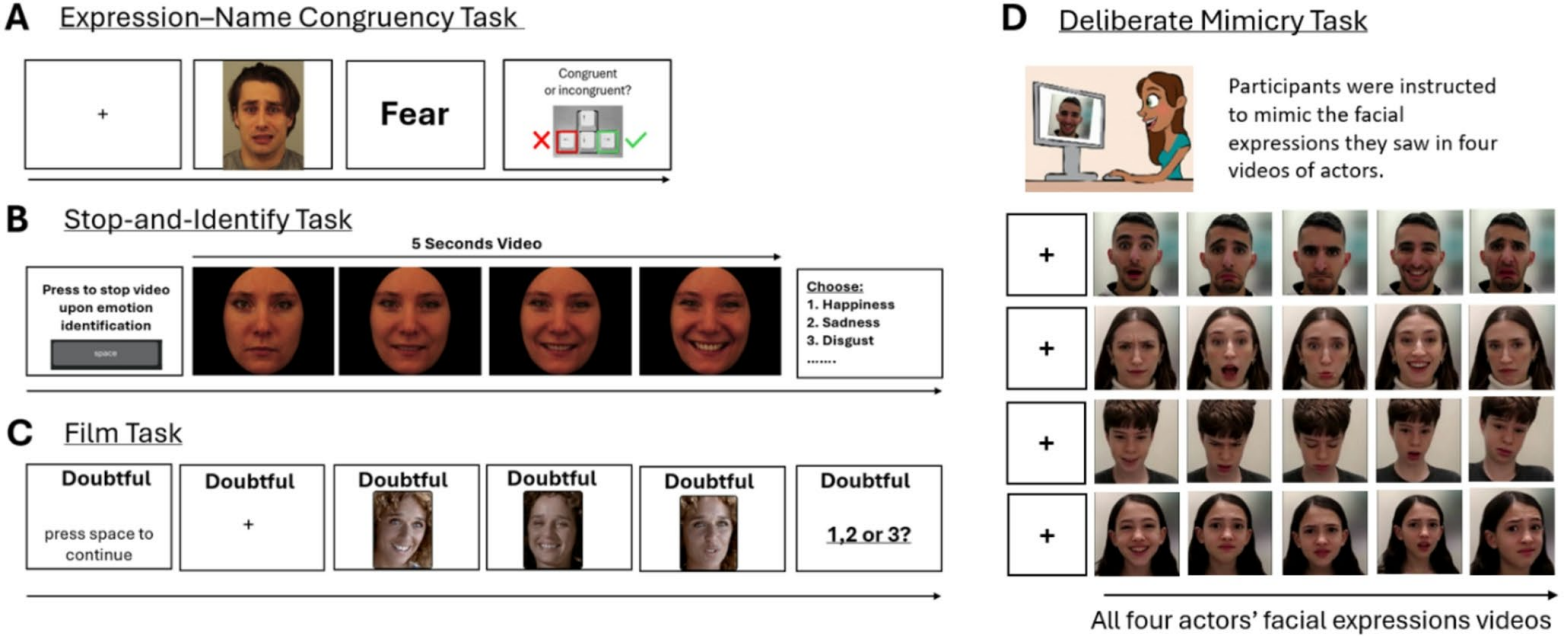
Tasks used to assess emotion-recognition performance. **A** Expression–Name Congruency. On each trial, a fixation cross preceded a face and an emotion word; participants judged whether the word matched the facial expression (congruent vs. incongruent). Accuracy and reaction time were recorded. **B** Stop-and-Identify. Participants viewed a 5 s dynamic face video and pressed the space bar as soon as they could identify the emotion, then selected the label from a list. Decision time (from video onset to keypress) and identification accuracy were recorded. **C** Film Task. Participants viewed naturalistic faces from film material and made a three-alternative forced-choice judgment for the target emotion (e.g., “Doubtful”). Accuracy was recorded. **D** Deliberate Mimicry task. participants were instructed to mimic the actors’ facial expression while being recorded using webcam

*Expression–Name Congruency Task -* adapted from Jospe et al., 2018, the task comprised 12 training trials and 105 congruent and 105 incongruent experiment trials, evenly distributed across the six basic emotions (happiness, sadness, anger, surprise, fear and disgust). On each trial, a fixation cross (1 × 1 cm; ~0.8°) appeared for 1 s followed by a still image of an emotional facial expression (15 × 15 cm; ~12.2°) presented for 400 ms, followed by an emotion label displayed for 400 ms. After the label disappeared, participants had up to 2 s to indicate whether the label matched the facial expression, using the left or right arrow keys. The key-response mapping (i.e., which key corresponded to “congruent” or “incongruent”) was randomly assigned across participants to control for response biases. Stimuli were randomly drawn from the Karolinska Directed Emotional Faces (KDEF) database (Lundqvist et al., 1998), counterbalanced for gender and emotion. Figure 1a displays a sample sequence of events for this task.

*Stop-and-Identify Task* - adapted from Heuer et al., 2010 and Calvo et al., 2018, this task consisted of 6 videos (15 × 15 cm; ~12.2°) for training and 72 videos (15 × 15 cm; ~12.2°) for the experiment, each lasting 5 s, depicting the six basic emotions. Half of the videos featured female expressers and half male expressers. Each video depicted a facial expression gradually transitioning from a neutral face to a fully expressed emotion. On each trial, participants were instructed to press the space bar to stop the video as soon as they recognized the emotion. Immediately afterward, they were asked to identify the emotion using the keyboard (1–6). Response time was recorded based on the moment the video was stopped (participant’s space bar key press), and accuracy was defined by correct emotion identification. Between each trial, an instruction screen appeared guiding the user to place his index finger on the space key and press space to continue. Trials with response times below 150ms were excluded to filter out accidental double-presses, which occurred in fewer than 0.02% of trials. Stimuli were adapted from the Karolinska Directed Emotional Faces – Dynamic (KDEF-dyn) database (Calvo et al., 2018). Since the original videos are 1 s in duration (30 frames), each frame was repeated five times to create a 5-second slow-motion version, allowing for more precise timing of emotion recognition. Figure 1b displays a sample sequence of events for this task.

*Film Task -* adapted from Garrido et al., 2009, this task involved presenting participants with 61 triplets of emotional facial expressions (9.5 × 11.5 cm; ~ 7.8° × 9.4°), of which 3 triplets were for the training trials. The stimuli consisted of realistic, ambiguous facial expressions captured from films and depicting complex emotional states beyond the basic emotions (e.g., contemptuous, awed, horrified, lustful), requiring participants to discriminate between subtle expressions that do not map onto prototypical basic emotion categories. Full details of the stimulus development and validation process are available in the supplementary materials of Garrido et al. (2009). Each triplet featured three different expressions portrayed by the same actor. On each trial, an emotional word describing the target state appeared at the top of the screen. Participants pressed the space bar when ready, after which the three facial expressions were presented sequentially, each for 1 s, while the emotion label remained visible throughout. After viewing the complete sequence, participants selected which image best matched the target emotion by pressing the corresponding key (1, 2, or 3) on the keyboard without time limitation. Figure 1c displays a sample sequence of events for this task.

#### Recognition Tasks Selection Rationale

Our three recognition tasks were selected to capture complementary facets of emotion recognition: rapid perceptual matching under time pressure (Expression–Name Congruency), dynamic detection of emerging expressions (Stop-and-Identify), and fine-grained discrimination among similar expressions (Film Task). This battery incorporates both static and dynamic stimuli, varied response demands, and - in the Film Task - genuinely ambiguous expressions drawn from naturalistic film scenes that require participants to discriminate between subtle, complex emotional states. However, we acknowledge that all three tasks present clearly visible, unoccluded faces. Theoretical accounts propose that sensorimotor simulation contributes most to recognition when visual emotion information is sparse or challenging - the more difficult an expression is to decode, the more simulation is recruited to compensate for limited or ambiguous perceptual input (Niedenthal et al., 2010; Wood et al., 2016). While the Film Task introduces interpretive ambiguity through subtle, non-prototypical expressions, our paradigm does not include conditions with visually degraded stimuli (e.g., low-intensity morphs, partial occlusion, or noise-masked faces) that would most strongly engage simulation mechanisms.

#### Mimicry Quality Assessment Task

*Deliberate Mimicry Task* - In this task, participants viewed four video clips (19.5 × 20.5 cm; ~15.9° × 16.7°; average length of 46 s) of individuals expressing five of the basic emotions (happiness, sadness, anger, surprise and disgust) and were instructed to mimic each facial expression as it was presented. Between each video there was a 5 s fixation cross (1 × 1 cm; ~0.8°). The stimuli were developed in-house and featured four different expressers: two adults, one male (27 years old) and one female (25 years old) and, to support generalizability two children: one boy (12 years old), and one girl (11 years old). Each video clip consisted of a single continuous recording of one expresser performing all five emotions in sequence, with each emotional expression lasting 3–6 s in a nearly fixed order that was highly similar across actors (A1: surprise-sadness-anger-happiness-disgust; A2: surprise-anger-disgust-sadness-happiness; A3 and A4: anger-happiness-sadness-surprise-disgust). While the presentation order of the four video clips was randomized across participants, the limited variability in within-actor emotion order is a methodological limitation addressed in Study 2. Participants were filmed via webcam (Logitech c930e; 1080p@30fps) at the exact time each trial was presented, capturing their facial responses in real time. This task did not consist of training trials, and the presentation order of the four video clips was randomized across participant. (See Fig. 1d).

The deliberate mimicry task was administered last to avoid priming participants with the concept of facial mimicry, which could have induced mimicry behavior during the emotion recognition tasks.

The deliberate mimicry task included five basic emotions (happiness, sadness, anger, surprise, and disgust). Fear was inadvertently omitted from the stimulus videos. While there is ongoing debate in the literature about which emotions qualify as “basic” (Tracy & Randles, 2011), and evidence suggesting that cross-cultural emotion communication may rely on fewer than six latent expressive patterns (Jack et al., 2016), this omission was not theoretically motivated but rather an oversight during stimulus development. Critically, because we average mimicry quality across emotions to capture general expressive ability rather than emotion-specific tendencies, the omission of fear from the mimicry task does not affect our primary conclusions.

#### Mimicry Accuracy Computation Algorithm

To compute mimicry accuracy and mimicry lag, we developed a method for comparing facial expressions between a presenter and a participant. We utilized a pretrained neural network (Savchenko et al., 2022; https://github.com/av-savchenko/hsemotion) trained to classify AffectNet 8 class emotional facial expressions (Mollahosseini et al., 2017). Rather than using the network’s final classification layer, we extracted the output from the penultimate feature layer—a 1408-dimensional embedding—which captures high-level expressive features.

Advances in deep learning have revealed that the rich, continuous feature spaces that emerge inside a neural network, before any class probabilities are computed, preserve the perceptual similarity structure humans rely on when judging complex stimuli (Zhang et al., 2018). Because these intermediate embeddings remain high-dimensional and unconstrained by the final classifier, they offer a principled basis for nuanced comparison. In the domain of facial identity, it was shown that distances measured in a network’s feature layer, capture the view-invariant critical features that underlie human face recognition (Abudarham et al., 2021) and are correlated with human similarity judgements (Shoham et al., 2024). Together, these findings justify our decision to extract vectors from the network’s penultimate layer, and use these representations vectors’ similarity to index facial expressions’ similarity: this approach preserves subtle differences in the embedding space that theoretically correspond to nuanced variations in facial expressions production.

For each frame in both the presenters’ and participants’ videos, we cropped a bounding box around the face using the MTCNN algorithm (Zhang et al., 2016). Each cropped frame was then passed through the pretrained neural network to obtain a 1408-dimensional embedding vector.

We focused our analysis on each of the emotions in the presenters’ videos: happiness, surprise, anger, sadness, and disgust. For each of these emotions, we identified a 3-second segment centered on the peak expression of that emotion. Segments in which either the participant or presenter had more than 20% missing face detections were excluded from analysis. Within each segment, we applied a summarization encoding to the temporal embedding data (Num_frames × Num_features matrix) by extracting four statistical features across time for each dimension: mean, maximum, minimum, and standard deviation (STAT encoding; Bargal et al., 2016). This encoding approach is specifically recommended for temporal video analysis with the emotion recognition architecture we employed (Savchenko, 2021; Savchenko et al., 2022). This yielded a 4×Num_features sized matrix, which was then flattened into a 1-dimentional representation vector.

**Fig. 2:**
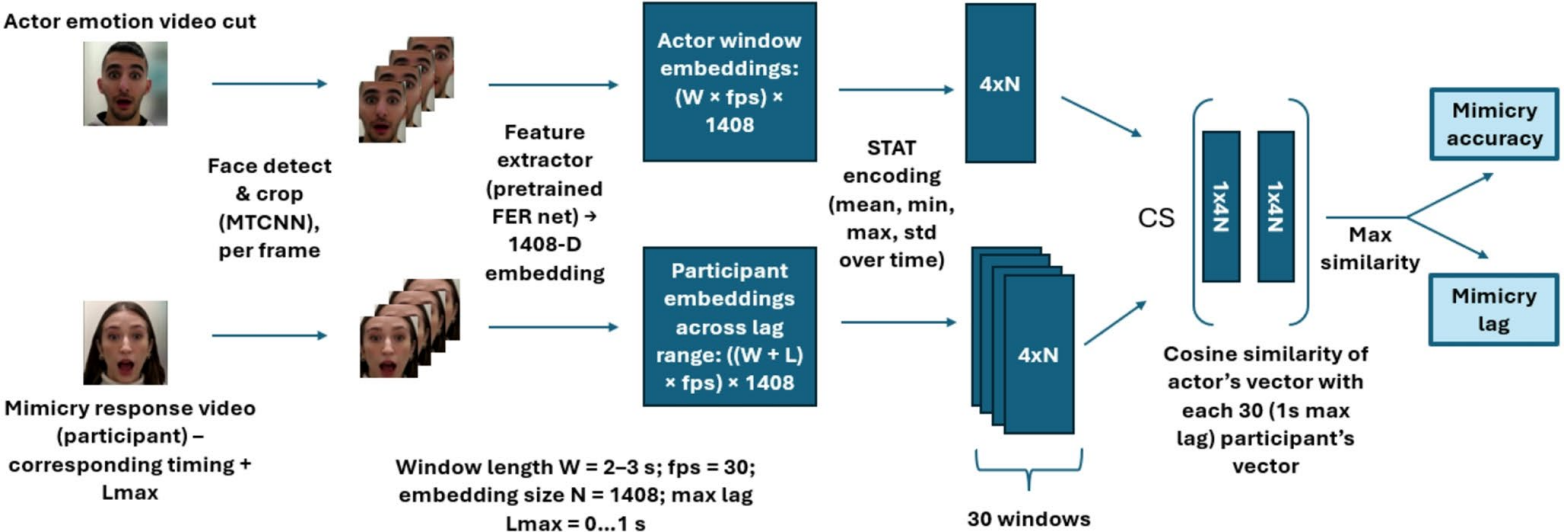
Pipeline for computing mimicry accuracy and mimicry lag from video recordings. The actor’s emotion video (top) and participant’s mimicry response video (bottom) are processed through identical preprocessing steps. Faces are detected and cropped per frame using MTCNN. Each cropped frame is passed through a pretrained FER network to extract 1408-dimensional embeddings from the penultimate layer. For the actor, a fixed 3-second window (W = 2–3 s; fps = 30) centered on each target emotion is processed. For the participant, a sliding window approach searches across temporal lags (W + L, where L ranges from 0 to 1 s) to identify optimal temporal alignment. Within each window, STAT encoding aggregates temporal dynamics by computing mean, minimum, maximum, and standard deviation across time for each feature dimension, producing 4×N flattened vectors. Cosine similarity is computed between the actor and participant STAT-encoded vectors across 30 possible lag offsets (one per frame in the 1-second lag range). The maximum cosine similarity across all lags yields mimicry accuracy, while the temporal offset at which this maximum occurs yields mimicry lag for each emotion window

To assess facial mimicry quality and lag, we used Windowed Time Lagged Cross-Correlation (WTLCC) analysis (Amihai et al., 2025; Riehle & Lincoln, 2018; Boker et al., 2002) with cosine similarity of STAT-encoded representation vectors as the between-windows comparison metric. For each 3-second emotion window in the presenter’s video, we applied a sliding window approach to the participant’s video, shifting frame by frame to allow for a maximum lag of 1 s. At each lag offset, we computed the cosine similarity between the presenter and participant STAT vectors. We identified the optimal lag as the temporal offset that yielded the highest similarity score for each emotion window. This WTLCC process resulted in two measures per emotion: mimicry accuracy, defined as the maximum similarity score across all lags, and mimicry lag, the temporal offset that produced the highest similarity between the participant and presenter for that specific emotion (See Fig. 2). WTLCC parameters (window size and maximum lag) choice justification is available at the Supplementary Materials (Supplemental section “WTLCC parameters”).

#### Algorithm Validation and Cutoff Determination

To ensure that our mimicry measurements reflect genuine facial mimicry, we established a validity threshold for the cosine similarity scores. Our algorithm generated similarity scores for each emotion × actor instance during the instructed-mimicry task. However, some scores may be uninformative due to technical issues (brief tracking failures, face occlusions) or instances where participants did not comply with task instructions. To prevent such invalid measurements from diluting our data, we applied a standard approach to define a validity threshold based on Youden’s index (Youden, 1950; Fluss et al., 2005); the full computation and validation procedure are described in the supplementary materials (Supplemental section “Algorithm Validation and Cutoff Determination”). Using this cutoff, we filtered out all emotion × actor mimicry accuracy and mimicry lag values for each participant if the mimicry accuracy value was below the threshold.

As we used Study 1 data for both algorithm validation and hypothesis testing, Study 2 serves as the critical confirmatory test: it applies the validated algorithm to a completely independent dataset that played no role in algorithm development or cutoff determination.

#### Algorithm Performance Validation

To showcase the algorithm’s ability to discriminate between different emotional expressions, we created a confusion matrix examining cross-emotion similarity patterns. For each participant and emotion, we computed similarity scores between participant’s facial expressions and actor’s representations of all five emotions—not just the target emotion. For example, when a participant mimicked happiness, we measured how similar their expression was to the actor’s happiness, but also to that same actor’s sadness, anger, surprise, and disgust. These similarity scores were averaged across participants and instances to create a 5 × 5 confusion matrix, where rows represent the emotion participants were instructed to mimic and columns represent the actor’s emotion used for comparison. This analysis serves to validate the algorithm’s emotion-specificity: if the algorithm works properly, we should see highest similarity values along the diagonal (when participant and actor emotions match) and systematic patterns of cross-emotion confusion consistent with psychological models of emotion similarity.

#### Mimicry Skill Reliability

To establish whether mimicry accuracy and mimicry lag function as a reliable skill, we computed intraclass correlation coefficients (ICCs) using a two-way random-effects model. Each participant was assessed across multiple actor×emotion combinations (4 actors × 5 emotions = 20 items), with each combination presented once. We report ICC(2,k), which quantifies the proportion of variance attributable to stable between-participant differences when both participants and items are random samples, reflecting absolute agreement across the k items (Shrout & Fleiss, 1979; McGraw & Wong, 1996). We also report ICC(3,k), which treats the specific 20 items as fixed and assesses consistency in participant scores (Koo & Li, 2016). Confidence intervals were computed using the F-distribution approximation in the psych package in R (Revelle, 2023).

#### Drift Diffusion Model

The drift-diffusion model (DDM) describes a rapid choice process as a noisy stream of evidence that begins from a starting point and accumulates until it reaches one of two decision boundaries (Ratcliff & McKoon, 2008). Its standard parameter set comprises the drift rate, boundary separation, starting point, and non-decision time. Boundary separation reflects the amount of evidence a decision-maker requires before committing to a response, with higher values indicating a more cautious, accuracy-oriented strategy independent of perceptual efficiency. Starting point marks any a-priori bias in the decision, and non-decision time captures motor execution and encoding processes unrelated to the decision itself. The parameter of central interest in the present work is the drift rate. Because drift rate measures the rate with which task-relevant information is sampled, a higher positive drift rate indicates that evidence for the correct alternative accrues more rapidly, reflecting both faster and more accurate decisions (Lerche & Voss, 2016; Ratcliff et al., 2016). This decomposition of response times and accuracy into underlying cognitive components has proven valuable in various facial emotion recognition tasks (Tipples, 2019; Lerche et al., 2021; Sawada et al., 2022).

When a task offers exactly two response options, researchers routinely fit the model to the distributions of correct versus incorrect reaction times, coding every error, regardless of the specific motor response as incorrect (Ratcliff & McKoon, 2008; Wagenmakers et al., 2007). The same accuracy-coding strategy has also proven effective in tasks with three or more options, where the single target option is treated as the “correct” boundary and all remaining options are pooled into one “error” boundary (Hedge et al., 2022; Schubert et al., 2016; Szul et al., 2020). This literature supports our use of drift rate as a direct index of task proficiency: higher drift rate values reflect a capacity to accumulate diagnostic evidence efficiently, yielding faster and more frequent correct responses.

#### Computing Drift-rate

We fitted hierarchical Bayesian drift–diffusion models using the R package brms (Bürkner, 2017) with the Wiener likelihood, using identity, log, and logit links for drift (v), boundary separation (a), nondecision time (Ter), and starting-point bias (w), respectively; correct responses were coded to the upper boundary. Priors (on the link scales) were weakly informative and task-appropriate, consistent with established DDM parameterizations and ranges (Ratcliff & McKoon, 2008; Ratcliff et al., 2016). Priors and appropriate prior predictive checks are available in the supplementary materials. Convergence of the MCMC draws was evaluated with the RHAT and bulk/tail effective sample size (ESS) diagnostics (Vehtari et al., 2021). Following that, each subject’s drift rate in each task was extracted. This drift rate captures the quality of evidence accumulation toward the correct decision, independent of any speed-accuracy tradeoff considerations. The drift rate parameter provides a process-pure measure of evidence accumulation efficiency in the emotion recognition tasks, complementing the proportion-correct analysis in assessing the link between deliberate facial mimicry quality and emotion recognition.

#### Statistics

We fitted two separate models: one with emotion-recognition accuracy as the outcome and another with drift rate as the outcome. For both models, this process yielded one observation per participant per task, resulting in three rows per participant corresponding to the three emotion-recognition tasks: the Expression–Name Congruency Task, the Stop-and-Identify Task, and the Film Task. For each participant, we computed two predictor variables from the Deliberate Mimicry Task: mimicry accuracy and mimicry lag. Both measures were averaged across presenters and emotions, excluding any instances where mimicry accuracy fell below the Youden-derived validity threshold, to characterize individual differences in general mimicry capacity and to maximize measurement reliability (four instances per emotion vs. twenty total). We used hierarchical mixed-effects models with random intercepts for both participants and tasks to capture general facial emotion-recognition ability rather than task-specific performance. This specification accounts for baseline ability differences between individuals while partialing out systematic difficulty differences between tasks, enabling inferences that generalize beyond the particular paradigms we sampled (Yarkoni, 2022; Barr et al., 2013; Baayen et al., 2008). This modeling strategy is further supported by evidence that emotion recognition reflects a general ability: prior work has shown that performance across facial, bodily, and vocal expression tasks is driven by a shared underlying skill (Connolly et al., 2020). In a similar way, our approach seeks to estimate a general ability within facial emotion recognition, combining performance across tasks that capture different aspects of recognizing emotions from faces.

To verify that mimicry accuracy and lag could be interpreted as independent predictors, we assessed multicollinearity using Variance Inflation Factors (VIF) and tested whether their effects were additive by examining their interaction (with predictors mean-centered). These diagnostic analyses are reported in the Supplementary Materials (Section: “Interaction Analyses of Mimicry Accuracy and Lag”).

Linear mixed-effects models implemented using lme4 package in R (Bates et al., 2015). For all models, effect sizes (e.g., Cohen’s f²) were computed using the effectsize R package, and both marginal and conditional R² values for mixed models were computed using the performance R package (Ben-Shachar et al., 2020; Lüdecke et al., 2021). The statistical significance threshold for all analyses was set at *p* < 0.05.

### Results

In this study we set out to test whether people who more precisely mimic facial expressions also recognize emotions more accurately and efficiently. We therefore modeled both (a) emotion recognition accuracy in each recognition task and (b) evidence-accumulation efficiency indexed by diffusion-model drift rate (v). Mixed-effects models (random intercepts for participants and tasks) tested mimicry accuracy and temporal lag from the Deliberate Mimicry Task as predictors.

#### Algorithm Performance Validation

To demonstrate the algorithm’s ability to discriminate between different emotional expressions, we created a confusion matrix examining cross-emotion similarity patterns. For each participant and emotion window, we computed similarity scores between the participant’s facial expressions and the actor’s representations of all five emotions—not just the target emotion. For example, when a participant mimicked happiness, we measured how similar their expression was to the actor’s happiness, but also to that same actor’s sadness, anger, surprise, and disgust. These similarity scores were averaged across all participants and instances.

**Fig. 3:**
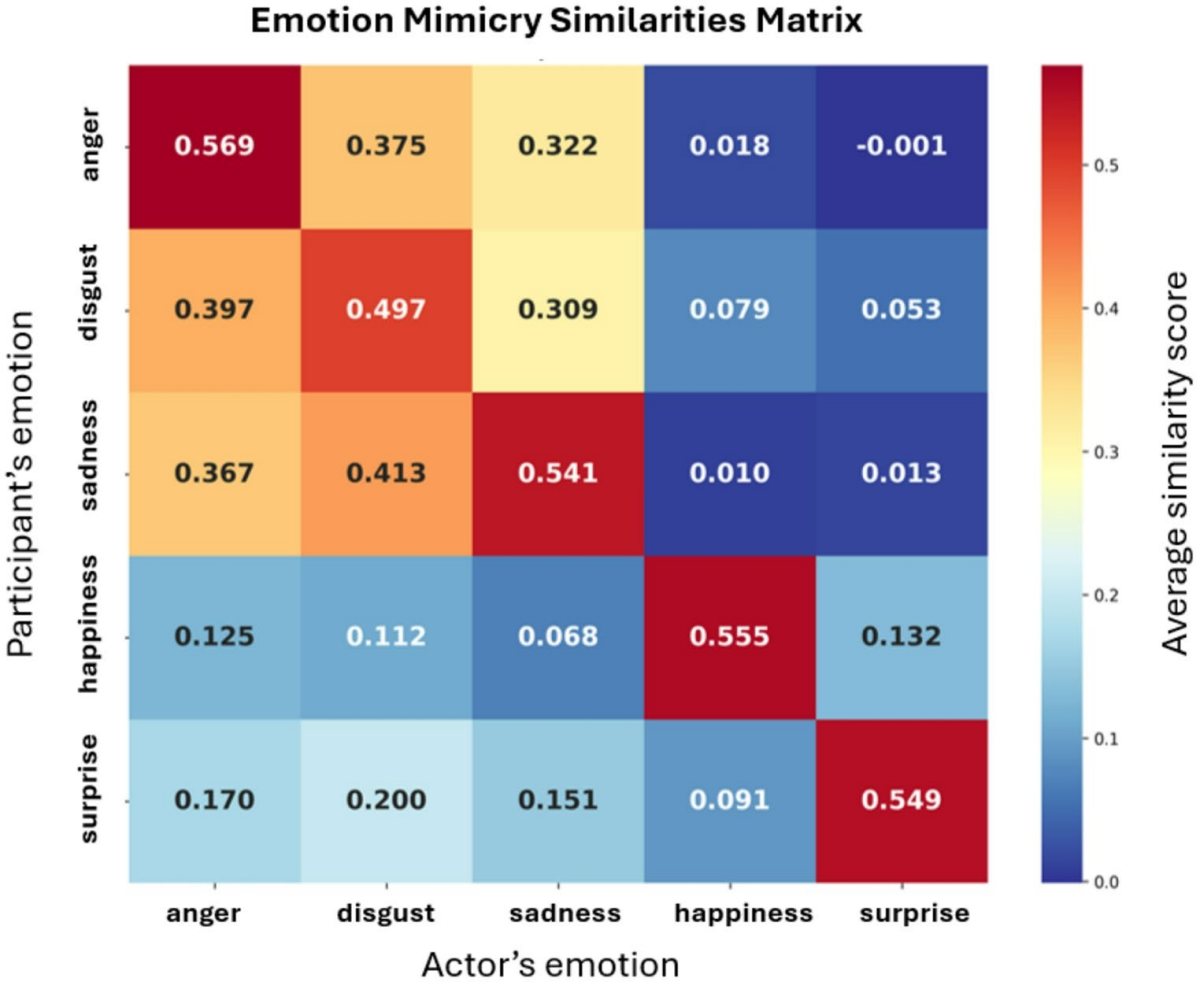
Emotion-specific mimicry patterns reveal dimensional structure of facial expressions. Confusion matrix showing average correlations between participants’ mimicked emotions (rows: what participants were instructed to mimic) and measured similarity scores across all five emotions (columns: algorithmic comparisons to each actor’s expressions). Strong diagonal values indicate emotion-specific mimicry accuracy. Off-diagonal patterns reveal systematic confusability: the three negative emotions (anger, disgust, sadness) show elevated cross-correlations, reflecting their proximity in valence-arousal space, while happiness and surprise remain more distinct. The matrix is asymmetric because correlations reflect directional comparisons—either participant mimicry of one emotion compared against all of that actor’s expressions, or one actor expression compared against all of the participant’s mimicked expressions—rather than pairwise emotion similarities. Plot created using Python Matplotlib (Hunter, 2007)

The confusion-matrix pattern (See Fig. 3) further validates the algorithm’s emotion-specific sensitivity: clear on-diagonal peaks with systematic cross-similarity among the three negatively valanced expressions (anger, sadness, disgust), alongside marked separations for happiness and for surprise (which does not cluster with happiness). This geometry accords with dimensional accounts in which perceived expression similarity is structured primarily by valence/arousal, yielding tight proximity among negative expressions and greater distance for happiness - as well as variable placement for surprise due to its ambiguous valence (Russell, 1980; Posner et al., 2005; Calvo & Nummenmaa, 2016; Young et al., 1997; Widen & Russell, 2010; Neta & Whalen, 2010).

#### Mimicry Skill Reliability

Mimicry accuracy demonstrated good reliability: ICC(2,k) = 0.74, 95% CI [0.60, 0.85]; ICC(3,k) = 0.77, 95% CI [0.65, 0.87] (*N* = 34 participants; 20 items). Mimicry lag showed moderate reliability: ICC(2,k) = 0.46, 95% CI [0.20, 0.68]; ICC(3,k) = 0.50, 95% CI [0.22, 0.72] (*N* = 34 participants; 20 items). These coefficients indicate that mimicry accuracy functions as a reproducible trait-like measure with good reliability, whereas lag exhibits modest but meaningful individual-level stability (interpretive benchmarks from Koo & Li, 2016).

#### Mimicry Accuracy and Mimicry Lag Predict Emotion Recognition Accuracy

For each emotion-recognition task, we calculated each participant’s emotion recognition accuracy. Mimicry accuracy and mimicry lag were derived from the deliberate mimicry task and averaged across emotions and actors for each participant. We then modeled emotion recognition accuracy with these two mimicry measures as predictors, including random intercepts for participant and task (102 observations: 34 participants × 3 tasks). Between-task correlations, and the correlations between mimicry accuracy and mimicry lag are reported in the supplementary materials (Supplemental Tables 1 and 3). Variance Inflation Factors indicated no multicollinearity concern (VIF = 1.00 for both predictors).

**Fig. 4:**
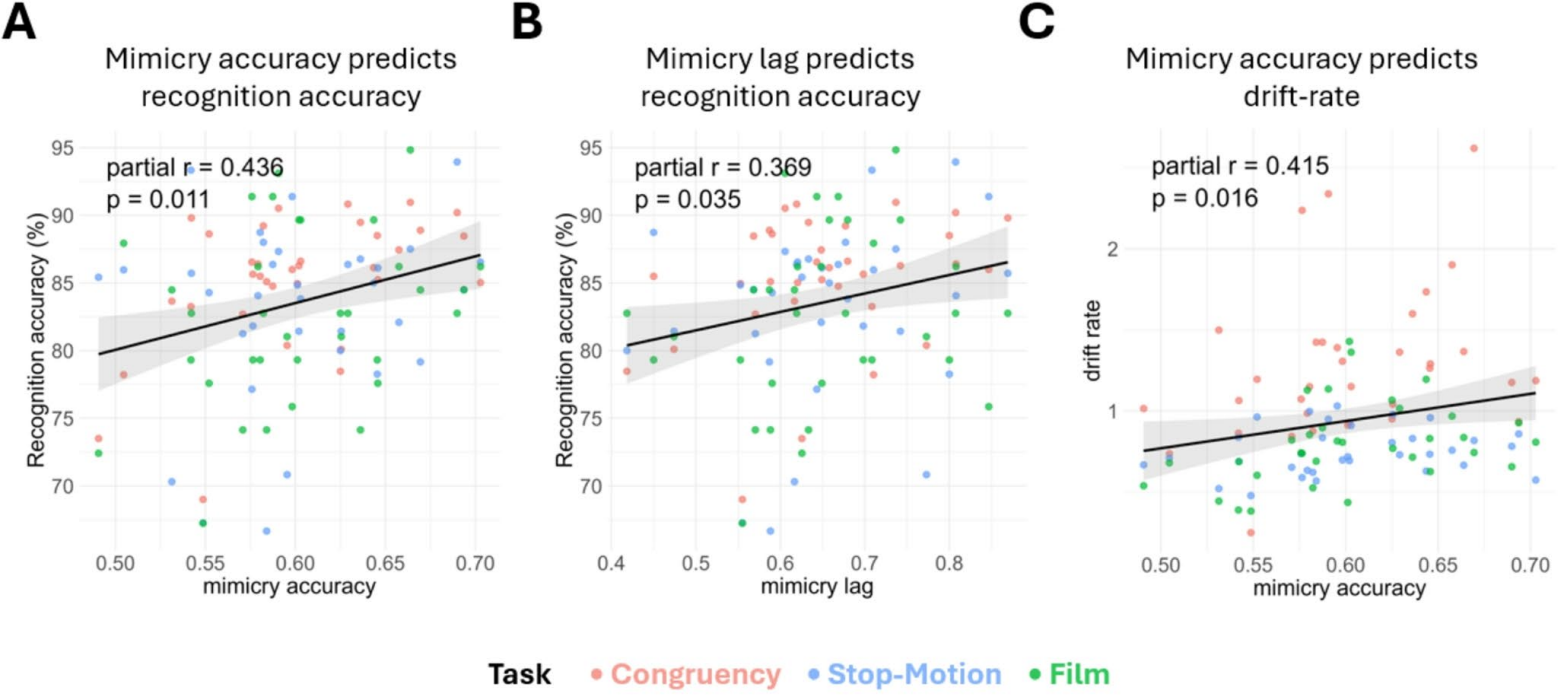
Relationship between mimicry metrics, recognition accuracy and drift-rate in study 1. **A** Mimicry accuracy was positively associated with recognition accuracy across tasks. **B** Mimicry lag also showed a positive association with recognition accuracy. **C** Mimicry accuracy is positively associated with drift rate across tasks. Lines are least-squares fits with 95% CIs within facets. Figures were created with ggplot2 (Wickham, 2016)

**Fig. 5:**
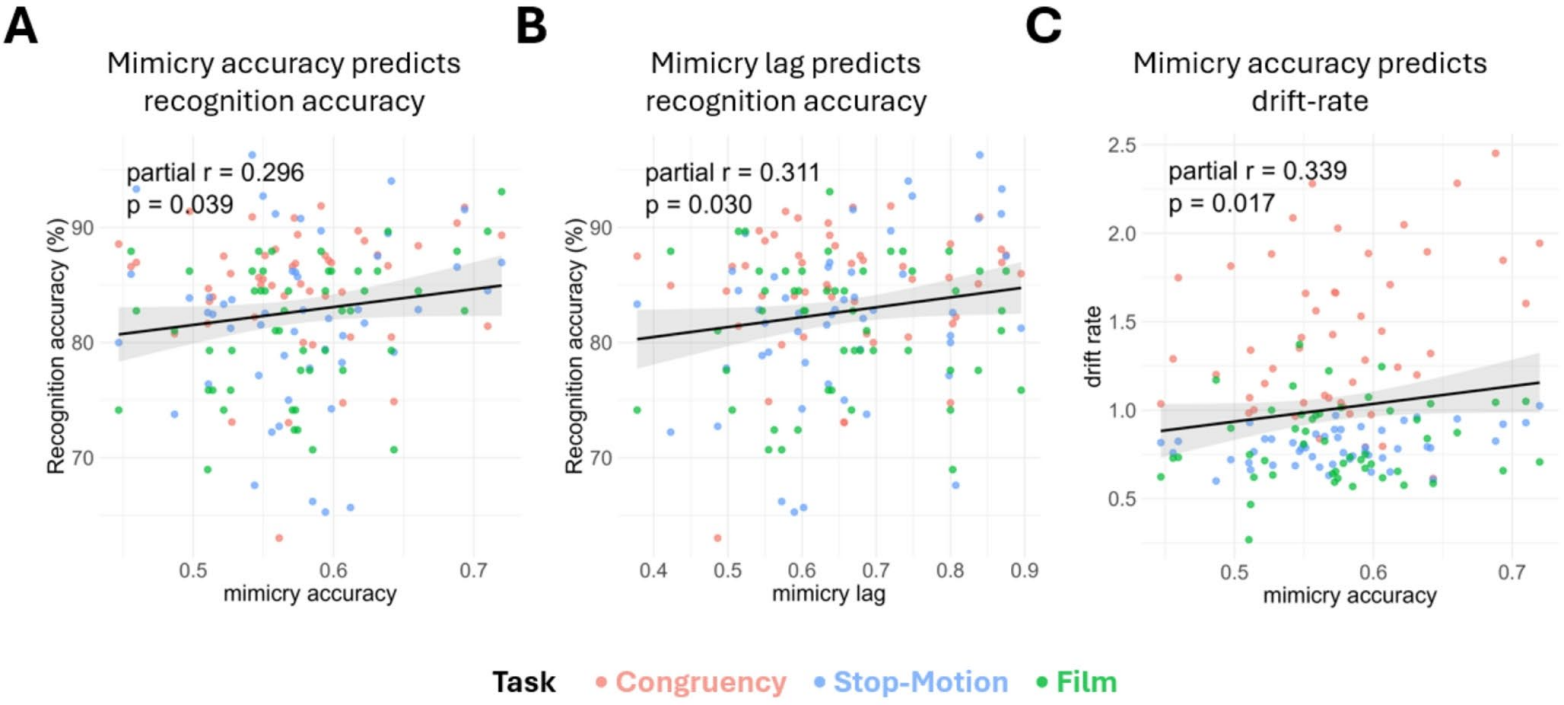
Relationship between mimicry metrics, recognition accuracy and drift-rate in study 2. **A** Mimicry accuracy was positively associated with recognition accuracy across tasks. **B** Mimicry lag also showed a positive association with recognition accuracy. **C** Mimicry accuracy is positively associated with drift rate across tasks. Lines are least-squares fits with 95% CIs within facets. Figures were created with ggplot2 (Wickham, 2016)

Both mimicry accuracy and mimicry lag significantly predicted emotion recognition performance. Mimicry accuracy positively predicted emotion recognition accuracy, b = 0.36 (95% CI[0.1, 0.71]), t(31) = 2.70, *p* = 0.011, Cohen’s f² = 0.23 (Fig. 5A), and mimicry lag also emerged as a significant predictor, b = 0.14 (95% CI[0.02, 0.27]), t(31) = 2.21, *p* = 0.035, Cohen’s f² = 0.16 (Fig. 5B). The fixed effects together explained 14.4% of the variance (marginal R²), with the full model accounting for 39.4% of variance when including random effects (conditional R²). These results suggest that both the accuracy and temporal dynamics of mimicry are positively associated with enhanced emotion recognition success (See Fig. 4A-B).

The hierarchical model revealed a consistent positive relationship between mimicry accuracy and emotion recognition performance across tasks as well as positive relationship between mimicry lag and emotion recognition performance. Task specific Pearson correlations demonstrated the same positive relationship (Mimicry accuracy - Congruency: *r* = 0.496, *p* = 0.003; Film: *r* = 0.276, *p* = 0.115; Stop-Motion: *r* = 0.188, *p* = 0.286; Mimicry lag - Congruency: *r* = 0.326, *p* = 0.06; Film: *r* = 0.192, *p* = 0.276; Stop-Motion: *r* = 0.236, *p* = 0.180) (See supplemental Fig. 2). Significance likely reflects the limited statistical power of separate analyses with smaller sample sizes (*N* = 34 per task) and task-specific measurement characteristics, whereas the hierarchical approach pools information across tasks to estimate the underlying relationship more stably and assess general emotion recognition capacity.

#### Mimicry Accuracy Predicts Drift-rate

To examine whether facial mimicry relates not only to recognition accuracy but also to the efficiency of evidence accumulation, we next analyzed drift rate parameters derived from the diffusion models. Convergence of the drift-diffusion models was evaluated via visual inspection of trace plots and the Gelman–Rubin R-hat. As recommended, R-hat values were very close to 1.00. All R-hat and effect sample size are reported in the supplementary materials.

We modeled participants’ drift-rate across the tasks with linear mixed-effects models (random intercepts for participant and task). Mimicry accuracy positively predicted drift rate, b = 1.68 (95% CI[0.39, 2.98]), t(31) = 2.54, *p* = 0.016, Cohen’s f² = 0.21; mimicry lag was not associated with drift rate, b = 0.07 (95% CI[-0.57, 0.70]), t(31) = 0.2, *p* = 0.840, Cohen’s f² = 0.01. The fixed effects explained 4.1% of the variance (marginal R²), with the full model accounting for 52.4% of variance when including random effects (conditional R²). These results suggest that higher mimicry accuracy is associated with stronger evidence accumulation toward correct emotion recognition responses (See Fig. 4C).

Consistent with the hierarchical model findings, task-specific correlations between mimicry accuracy and drift-rate showed similar positive patterns (Congruency: *r* = 0.316, *p* = 0.069; Film: *r* = 0.345, *p* = 0.046; Stop-Motion: *r* = 0.178, *p* = 0.314), though with varying significance likely due to reduced power in separate analyses (See supplemental Fig. 3).

## Study 2

Building on Study 1’s findings that mimicry capabilities can predict participant’s emotion recognition abilities, Study 2 was designed as a pre-registered replication with expanded hypotheses ( https://aspredicted.org/4v47-227x.pdf ).

We first sought to replicate Study 1’s core findings: that (i) mimicry accuracy would be positively associated with both the emotion recognition tasks’ accuracy and drift rate, and (ii) that mimicry lag would be positively associated with emotion recognition accuracy.

Furthermore, we preregistered the hypothesis that higher mimicry quality and FER performance would be associated with higher empathy (IRI) and lower autistic traits (AQ). We examined correlations between these questionnaires and our primary measures, and assessed whether empathy and autistic traits would account for the mimicry-recognition relationship by including them as model predictors. These predictions were informed by findings that mimicry is modulated by empathic capacity (Drimalla et al., 2019), diminished in autism (McIntosh et al., 2006), and that elevated autistic traits weaken the mimicry-recognition relationship (Folz et al., 2023).

### Method

#### Sample Size and Power Analysis

Study 2 was designed to have adequate power to replicate the core mimicry-recognition relationship observed in Study 1. In the prior study, mimicry accuracy demonstrated a medium-sized partial effect on recognition outcomes (Cohen’s f² = 0.21–0.23; equivalent *r* ≈ 0.41–0.43). Based on Fisher’s z transformation (two-tailed test, α = 0.05), detecting an effect of *r* = 0.41 requires approximately *N* = 45 participants to achieve 80% power. We therefore set a target sample size of *N* = 50 to maintain at least 80% power after accounting for potential participant exclusions.

Importantly, this sample size was determined solely for replicating the primary mimicry-recognition relationship and was not powered to detect associations with the additional questionnaire measures (AQ, IRI). Detecting small-to-medium correlations between questionnaires and our primary measures would require substantially larger samples. The questionnaire analyses should therefore be interpreted as exploratory, and null findings do not rule out potentially meaningful associations that a larger sample might detect.

#### Participants

Fifty participants (13 males and 37 females), aged 19 to 44 years (*M* = 23.38,* SD* = 3.49), were recruited from the same university population as Study 1. The study was approved by the university’s ethics committee, and all participants provided written informed consent and received monetary compensation or course credit for their time.

#### Procedure

In addition to the Expression–Name Congruency Task, Stop-and-Identify Task, Film Task, and Deliberate Mimicry Task described in Study 1, participants also completed the Reading the Mind in the Eyes Task (RMET; Baron-Cohen, Wheelwright, Hill et al., 2001; Baron-Cohen, Wheelwright, Skinner et al., 2001) and two self-report questionnaires.

*The RMET* consists of 36 black-and-white photographs (12.5 × 5 cm; ~ 10.2° × 4.1) showing only the eye region of different individuals, each conveying a subtle emotional or mental state. On each trial, participants are presented with four words describing distinct emotional states and are asked to select the one that best matches the expression in the image. Each item has a single correct answer, and no feedback is provided throughout the task. After each trial, participants were asked to rate their confidence in their response on a 1–9 scale. Because RMET accuracy can be constrained by stimulus and lexical demands, confidence ratings provide a complementary window onto the subjective strength of evidence guiding social judgments (Fleming, 2024). Empirical RMET work shows confidence can systematically diverge from accuracy - including overconfidence on incorrect items - making it a sensitive marker of social metacognition (Schilling et al., 2012; Cyrkot et al., 2021).

*Deliberate Mimicry Task* – In this study, we introduced three modifications to the previously described deliberate mimicry task. The first modification involved adding a calibration phase in which participants were filmed while performing two calibrations consisting of multiple mouse clicks. At the beginning of the task, they clicked on nine red dots distributed across the screen, with both click timing and dot location recorded. Before each video trial, participants clicked on 12 red dots positioned around the approximate locations of the eyes and mouth of the upcoming presenter. This procedure was designed to approximate participants’ eye gaze behavior. Eye gaze analysis was beyond the scope of the current study, which focused specifically on the relationship between facial mimicry and emotion recognition performance. However, this data was collected to support future investigations into the role of gaze patterns in facial mimicry processes.

The second modification involved updating the stimulus videos to create a more controlled filming environment, with standardized filming location, camera setup, and lighting conditions. We used the same actors as in Study 1. In these new recordings, the actors returned to a neutral expression after displaying each emotional expression, whereas in the original videos actors transitioned directly from one emotion to the next without neutral expressions in between.

The third modification involved improvement of the emotion ordering relative to Study 1, which had a nearly fixed emotion order across actors. Study 2 used four fully distinct randomized orders across actors (A1: surprise-sadness-anger-happiness-disgust; A2: anger-surprise-sadness-happiness-disgust; A3: happiness-sadness-surprise-anger-disgust; A4: disgust-happiness-anger-sadness-surprise), ensuring that no emotion was systematically presented at the same serial position. To assess individual differences in socio-emotional traits, participants also completed the Interpersonal Reactivity Index (IRI; Davis, 1983), a measure of subjective empathy that captures variation in social sensitivity and emotional responsiveness, and the Autism-Spectrum Quotient (AQ; Baron-Cohen, Wheelwright, Hill et al., 2001; Baron-Cohen, Wheelwright, Skinner et al., 2001) included due to prior evidence linking autistic traits to reduced facial mimicry, which may explain variability in mimicry responses (McIntosh et al., 2006).

#### Computing Drift-rate

For Study 2, we carried out sequential Bayesian updating: the posterior from Study 1 was used as the prior for the same Wiener DDM fit in brms (Bürkner, 2017).

#### Exploratory Post-Hoc Analyses

To further elucidate the mechanisms underlying the mimicry–recognition relationship, we conducted two sets of exploratory analyses that were not preregistered. Both analyses combined data from Study 1 and Study 2 (*N* = 84 total), providing statistical power not available within each study separately.

##### Mimicry Lag and Decision Strategy

Our primary models revealed that mimicry lag predicted recognition accuracy but not drift rate - a dissociation suggesting lag operates through mechanisms other than perceptual efficiency. To further clarify the nature of this relationship, we examined whether mimicry metrics relate to other DDM parameters: boundary separation, which indexes the amount of evidence required before committing to a response (i.e., decision caution), and non-decision time, which captures motor execution and encoding processes unrelated to the decision itself. To obtain reliable individual-level estimates of boundary separation and non-decision time, we refitted the drift-diffusion models on the combined dataset (*N* = 84). 

Critically, this larger trial count enabled us to include random intercepts for participants on boundary separation and non-decision time parameters - random effects that could not be estimated reliably within each study separately due to insufficient trial counts. We then modeled each DDM parameter separately as outcomes in hierarchical mixed-effects models (random intercepts for participant and task), testing mimicry accuracy and mimicry lag as predictors in separate models.

##### Emotion-specific Mimicry–recognition Relationships

As a supplementary exploration, we tested whether mimicry accuracy for individual emotions predicts recognition performance for those same emotions, both for recognition accuracy and drift-rate outcomes. Given that each emotion yielded only four mimicry instances per participant, these emotion-specific estimates are noisier than our aggregated measure and should be interpreted with caution. Full methods and results are reported in the supplementary materials.

### Results

Study 2 was a preregistered replication of Study 1. We sought to replicate the association between deliberate mimicry quality and facial-expression recognition proficiency. Accordingly, we modeled both (a) emotion recognition accuracy in each task and (b) evidence-accumulation efficiency indexed by diffusion-model drift rate. Mixed-effects models with random intercepts for participants and tasks tested mimicry accuracy and temporal lag from the Deliberate Mimicry Task as predictors. We then extended the models to include individual-difference measures (gender, AQ, IRI) and, in parallel, examined performance on the RMET. Between-task correlations, and the correlations between mimicry accuracy and mimicry lag are reported in the supplementary materials (Supplemental Tables 2 and 4).

#### Mimicry Skill Reliability

Mimicry accuracy showed moderate-to-good reliability: ICC(2,k) = 0.59, 95% CI [0.43, 0.73]; ICC(3,k) = 0.67, 95% CI [0.52, 0.79]. Mimicry lag reliability improved relative to Study 1: ICC(2,k) = 0.65, 95% CI [0.50, 0.77]; ICC(3,k) = 0.71, 95% CI [0.58, 0.82]. The enhanced stability of both metrics in Study 2, likely reflects the methodological refinement of including neutral expressions between emotions in the stimulus videos, providing clearer temporal boundaries for detecting mimicry onset and thereby enabling more consistent measurement.

#### Mimicry Accuracy and Mimicry Lag Predict Emotion Recognition Accuracy

We began by testing the main hypothesis that mimicry accuracy and mimicry lag are positively associated with emotion recognition accuracy of the emotion recognition tasks. Participant’s emotion recognition accuracy across tasks were modeled as a function of mimicry accuracy and lag, with random intercepts for participant and task (150 observations from 50 participants across three tasks). Variance Inflation Factors indicated no multicollinearity concern (VIF = 1.07 for both predictors).

Both mimicry accuracy and lag significantly predicted emotion recognition performance. Mimicry accuracy positively predicted FER tasks proportion-correct, b = 0.21 (95% CI[0.02, 0.41]), t(47) = 2.12, *p* = 0.039, Cohen’s f² = 0.10 (Fig. 5A), and mimicry lag also emerged as a significant predictor, b = 0.11 (95% CI[0.01, 0.21]), t(47) = 2.24, *p* = 0.030, Cohen’s f² = 0.11 (Fig. 5B). The fixed effects together explained 6.4% of the variance (marginal R²), with the full model accounting for 31.2% of variance when including random effects (conditional R²). These results replicate Study 1’s findings, demonstrating that both the accuracy and temporal dynamics of mimicry are associated with enhanced emotion recognition performance (See Fig. 5A-B).

The hierarchical model revealed a consistent positive relationship between mimicry accuracy and emotion recognition performance across tasks as well as positive relationship between mimicry lag and emotion recognition performance. Task specific Pearson correlations demonstrated positive relationship (Mimicry accuracy - Congruency: *r* = 0.061, *p* = 0.672; Film: *r* = 0.324, *p* = 0.022; Stop-Motion: *r* = 0.091, *p* = 0.532; Mimicry lag - Congruency: *r* = 0.110, *p* = 0.446; Film: *r* = 0.003, *p* = 0.985; Stop-Motion: *r* = 0.348, *p* = 0.013), for mimicry accuracy mainly in the film task, and for mimicry lag mainly in the Stop-Motion task. Significance in task specific correlations likely reflects the limited statistical power of separate analyses with smaller sample sizes (*N* = 50 per task) and task-specific measurement characteristics, whereas the hierarchical approach pools information across tasks to estimate the underlying relationship more stably and assess general emotion recognition capacity (See supplemental Fig. 4).

#### Mimicry Accuracy Predicts Drift-rate

To test the hypothesis that mimicry accuracy is related to the efficiency of evidence accumulation, we next analyzed drift rate parameters derived from the diffusion models. Convergence of the drift-diffusion models was evaluated via visual inspection of trace plots and the Gelman–Rubin R-hat. As recommended, R-hat values were very close to 1.00. All R-hat and effect sample size are reported in the supplementary materials.

We modeled participants’ drift-rate across the tasks with linear mixed-effects models (random intercepts for participant and task) Mimicry accuracy positively predicted drift rate, b = 1.10 (95% CI[0.23, 1.97]), t(47) = 2.47, *p* = 0.017, Cohen’s f² = 0.13; mimicry lag was not associated with drift rate, b = 0.19 (95% CI[-0.25, 0.63]), t(47) = 0.85, *p* = 0.400, Cohen’s f² = 0.02. The fixed effects explained 2.0% of the variance (marginal R²), with the full model accounting for 66.3% of variance when including random effects (conditional R²). These results replicate Study 1’s findings and suggest that mimicry accuracy is associated with stronger evidence accumulation toward correct emotion recognition responses (See Fig. 5C).

Consistent with the hierarchical model findings, task-specific correlations between mimicry accuracy and drift-rate showed similar positive patterns (Congruency: *r* = 0.285, *p* = 0.045; Film: *r* = 0.102, *p* = 0.480; Stop-Motion: *r* = 0.361, *p* = 0.010), though with varying significance likely due to reduced power in separate analyses (See supplemental Fig. 5).

#### Questionnaire Correlations

Associations between questionnaire measures and study variables were largely non-significant, with one notable exception. AQ scores were positively correlated with mimicry lag (*r* = 0.30, 95% CI[0.03, 0.53], *p* = 0.033), indicating that individuals with higher autistic traits showed slightly longer temporal delays in their mimicry responses. Other correlations with the Autism-Spectrum Quotient were not significant: mimicry accuracy (*r* = -0.13, 95% CI[-0.40, 0.15], *p* = 0.356), mean drift rate (*r* = 0.03, 95% CI[-0.25, 0.31], *p* = 0.836), and mean accuracy (*r* = 0.09, 95% CI[-0.20, 0.36], *p* = 0.544). For the Interpersonal Reactivity Index, which measures self-reported empathy, correlations with mimicry accuracy (*r* = -0.04, 95% CI[-0.31, 0.24], *p* = 0.784), mimicry lag (*r* = -0.01, 95% CI[-0.29, 0.27], *p* = 0.930), mean drift rate (*r* = -0.20, 95% CI[-0.45, 0.08], *p* = 0.162), and mean accuracy (*r* = 0.04, 95% CI[-0.24, 0.31], *p* = 0.799) were not significant.

To examine whether self-reported empathy and autistic traits accounted for the relationship between mimicry and emotion recognition accuracy, we extended the base model by adding AQ and IRI total scores as predictors. When controlling for both questionnaires, mimicry accuracy (b = 0.22, 95% CI[0.02, 0.41], t(45) = 2.11, *p* = 0.041, Cohen’s f² = 0.10) and mimicry lag (b = 0.11, 95% CI[0.01, 0.21], t(45) = 2.05, *p* = 0.046, Cohen’s f² = 0.09) remained significant predictors. In contrast, neither AQ scores (b < 0.001, 95% CI[-0.002, 0.003], t(45) = 0.24, *p* = 0.810, Cohen’s f² < 0.01) nor IRI scores (b < 0.001, 95% CI[-0.001, 0.002], t(45) = 0.38, *p* = 0.707, Cohen’s f² < 0.01) significantly predicted accuracy. A likelihood ratio test confirmed that adding these questionnaire measures did not improve model fit, χ²(2) = 0.22, *p* = 0.894. These results suggest that the relationship between mimicry dynamics and emotion recognition is independent of self-reported empathy and autistic traits.

To examine whether self-reported empathy and autistic traits accounted for the mimicry-drift rate relationship, we added AQ and IRI scores to the base model. Results paralleled those for recognition accuracy: mimicry accuracy remained a significant predictor (b = 1.08, 95% CI[0.23, 1.94], t(45) = 2.42, *p* = 0.020, Cohen’s f² = 0.13), and mimicry lag remained non-significant (b = 0.16, 95% CI[-0.29, 0.61], t(45) = 0.69, *p* = 0.493, Cohen’s f² = 0.01). Neither AQ scores (b = 0.002, 95% CI[-0.008, 0.011], t(45) = 0.33, *p* = 0.740, Cohen’s f² < 0.01) nor IRI scores (b = -0.005, 95% CI[-0.011, 0.002], t(45) = -1.36, *p* = 0.181, Cohen’s f² = 0.04) significantly predicted drift rate. A likelihood ratio test confirmed that adding these questionnaire measures did not improve model fit, χ²(2) = 2.07, *p* = 0.355.

#### Reading the Mind in the Eye

While neither mimicry measures nor AQ predicted RMET accuracy or drift rate, mimicry accuracy was associated with higher confidence ratings in RMET responses (see supplementary materials for detailed analyses).

#### Exploratory Post-Hoc Analyses

To further clarify the mechanism through which mimicry lag relates to recognition performance, we examined its associations with boundary separation and non-decision time - DDM parameters that index decision caution and motor/encoding processes, respectively. Because reliable individual-level estimates of these parameters require sufficient trial counts, we conducted this analysis on the combined dataset (*N* = 84), fitting separate hierarchical mixed-effects models (random intercepts for participant and task) for each parameter. The DDM models converged successfully; convergence diagnostics (R-hat values and effective sample sizes) are reported in Supplementary Materials.

Mimicry lag significantly predicted boundary separation, *b* = 0.20 (95% CI [0.02, 0.37]), *t*(82) = 2.18, *p* = 0.032, *f²* = 0.06, but not non-decision time, *b* = -0.21 (95% CI [-0.68, 0.27]), *t*(82) = -0.87, *p* = 0.390, *f²* < 0.01. Consistent with the study-specific analyses, mimicry accuracy predicted drift rate in the combined sample, *b* = 0.91 (95% CI [0.40, 1.42]), *t*(82) = 3.48, *p* < 0.001, *f²* = 0.15, while mimicry lag did not, *b* = 0.17 (95% CI [-0.11, 0.45]), *t*(82) = 1.17, *p* = 0.246, *f²* = 0.02. Mimicry accuracy showed no association with either boundary separation, *b* = -0.18 (95% CI [-0.54, 0.17]), *t*(82) = -1.02, *p* = 0.310, *f²* = 0.01, or non-decision time, *b* = 0.16 (95% CI [-0.78, 1.09]), *t*(82) = 0.34, *p* = 0.736, *f²* < 0.01. This dissociation confirms that the two mimicry dimensions operate through distinct pathways: mimicry accuracy relates to perceptual efficiency (drift rate), while mimicry lag relates to decision strategy (boundary separation). This pattern suggests that individuals with longer mimicry latencies may set wider decision boundaries during emotion recognition, achieving higher accuracy through increased caution rather than enhanced evidence quality.

### Discussion

The present study began with a straightforward question: are individual differences in deliberate facial mimicry quality associated with the ability to recognize facial expressions? We tested this question across two independent samples and found that higher mimicry accuracy predicted better emotion recognition, as reflected in performance accuracy and drift-rate in facial emotion recognition tasks.

People spontaneously mimic observed facial expressions. Interfering with mimicry can affect emotion recognition—reducing facial movement impairs it, enhancing it helps (Neal & Chartrand, 2011; Oberman et al., 2007; Rychlowska et al., 2014; Borgomaneri et al., 2020). However, these effects vary by task and context (Hess & Fischer, 2013; Perugia et al., 2021), and the mimicry-recognition link remains inconsistent (Holland et al., 2021). Most previous studies measured mimicry and recognition concurrently, intertwining motor and decision processes. Prior work that measured mimicry quality, did so to self-imitated expressions (Cook et al., 2013), related it to non-recognition outcomes like empathy (Williams et al., 2013), measured mimicry quality concurrently during emotion recognition tasks and against still images (Drimalla et al., 2021), or treated mimicry as secondary to a recognition task (Perugia et al., 2021). Prior to this work, no studies had established facial mimicry quality as a trait—separately from emotion recognition—and tested whether individual differences in mimicry skill predict recognition performance.

Treating mimicry as a skill is theoretically valuable because it reveals potential double dissociations: poor mimicry with poor recognition suggests simulation difficulties, while good mimicry with poor recognition suggests downstream problems mapping emotion labels. Such dissociations appear in alexithymia, autism spectrum conditions, and Parkinson's disease, where mimicry and recognition are differentially affected (Franz et al., 2021; Nordmann et al., 2021; Parker et al., 1993). Clinically, someone struggling with mimicry timing and accuracy may require a different intervention than someone who mimics well but cannot label the emotion.

Embodied-simulation theories propose that observers sometimes recognize facial expressions by mapping them onto their own sensorimotor and affective systems, often instantiated as facial mimicry, although such simulation is not assumed to be necessary in all contexts (Hess & Fischer, 2024; Niedenthal et al., 2010; Wood et al., 2016). Rather than demonstrating that online mimicry is required for recognition on each trial, our findings show that individuals with higher-quality facial mimicry - possibly a marker of richer and more precise sensorimotor representations - also exhibit superior facial-expression perception. This pattern suggests that expressive expertise shapes emotion perception and parallels evidence from other action domains, where motor expertise predicts more accurate perception of those same actions (Aglioti et al., 2008; Calvo-Merino et al., 2010; Casile & Giese, 2006; Jospe et al., 2017, 2020; Ludolph et al., 2017; Repetto et al., 2021; Valchev et al., 2015).

Methodologically, we introduced and validated a computational approach for quantifying facial mimicry quality. Our algorithm uses high-dimensional feature embeddings from a pretrained emotion-recognition network and Windowed Time Lagged Cross Correlation, operating directly on naturalistic video recordings without specialized equipment. This approach captures holistic facial patterns without predefined regions of interest or action units. The ability to decompose mimicry into both accuracy and temporal lag components provides a richer characterization of individual differences, revealing that these two dimensions independently contribute to emotion recognition performance.

In facial-expression recognition research, accuracy and reaction time are standard performance measures, but they combine distinct cognitive processes: the quality of evidence accumulation, strategic response caution, and motor execution speed. The drift-diffusion model decomposes these components, with drift rate specifically capturing how efficiently diagnostic information is extracted from emotional faces - independent of decision strategies or peripheral processes (Ratcliff & McKoon, 2008; Ratcliff et al., 2016). In the present study, this decomposition was critical for understanding how different facets of mimicry relate to emotion recognition. Mimicry accuracy predicted both emotion recognition accuracy and drift rate, indicating that individuals who reproduce expressions more precisely also accumulate perceptual evidence more efficiently.

In contrast, mimicry lag predicted emotion recognition accuracy but showed no relationship with drift rate. This dissociation suggests that slower, more deliberate mimicry relates to recognition through mechanisms other than enhanced evidence accumulation. Several accounts may explain this pattern: longer mimicry latencies may reflect a cautious, accuracy-oriented processing style; delayed mimicry may index holistic processing where participants withhold their motor response until integrating the entire facial configuration - as diagnostic facial signals may emerge later in expression dynamics (Jack et al., 2014); or slower mimicry might reflect more deliberate perceptual encoding before reproduction—consistent with mimicry's context dependence (Hess & Fischer, 2024). Our exploratory combined-cohort analysis provides initial support for the cautious-responding account: mimicry lag was associated with higher boundary separation but not non-decision time, suggesting that individuals with slower deliberate mimicry responses set wider decision thresholds during recognition, achieving accuracy through increased caution rather than enhanced evidence quality. This finding, however, is exploratory and requires confirmation in studies specifically designed to investigate mimicry temporal dynamics.

Additionally, we observed that the relationship between mimicry lag and mimicry accuracy varied across emotions, ranging from positive (e.g., sadness) to negative (e.g., happiness; see Supplementary Table 4). This variability suggests that the temporal and spatial dimensions of mimicry may operate differently across emotions - for instance, some emotions may benefit from rapid mimicry while others require more deliberate processing. This pattern motivated exploratory emotion-specific analyses (N = 84; full methods and results in supplementary materials), which revealed that anger mimicry predicted anger recognition accuracy, and both anger and happiness mimicry were associated with emotion-specific drift rates, while other emotions showed weak or null effects. Given that our mimicry task included only four instances per emotion per participant, future research with more repetitions per emotion would be better positioned to reliably characterize these relationships.

We observed a specific association between autistic traits and mimicry temporal dynamics: individuals with higher AQ scores showed longer mimicry lags, whereas AQ scores were unrelated to mimicry accuracy, emotion-recognition accuracy, or drift rate. This pattern suggests that autistic traits may affect the timing of mimicry responses rather than mimicry quality or recognition ability and may relate to broader timing differences in mimicry reported in autism research (Drimalla et al., 2021; Oberman et al., 2009; Ruzich et al., 2015).

#### Limitations

First, the correlational design cannot establish whether superior mimicry directly enhances recognition, whether better recognition enhances mimicry, or whether shared factors contribute to both. While our study was designed to test whether representational richness predicts recognition ability, our findings do not demonstrate that online simulation occurs during recognition itself, nor can they rule out that the observed associations reflect general visuomotor skill, attentional engagement, or perceptual sensitivity. Second, we measured deliberate rather than spontaneous mimicry, which engages effortful visuomotor pattern matching and motor planning differently than the natural mimicry typically implicated in embodied-simulation theories. Third, although our sample size provided adequate power for the primary mimicry–recognition relationship, it offered limited power for detecting associations with questionnaire measures. The largely null findings relating AQ and IRI to mimicry and recognition should therefore be interpreted cautiously, as potentially meaningful associations may exist that our sample was underpowered to detect. Fourth, our mimicry task included only four instances per emotion (one per actor), providing relatively unstable emotion-specific estimates compared to our aggregated measure; nonetheless, exploratory analyses revealed that anger and happiness mimicry accuracy predicted recognition performance for those same emotions, a pattern worth pursuing in future research with more repetitions per emotion.

#### Future Directions

Several directions emerge from the current findings. First, testing whether facial mimicry quality predicts emotion recognition beyond the face would help distinguish domain-general embodied simulation from face-specific pattern matching. If our findings reflect embodied simulation more broadly, individuals with higher mimicry accuracy should also show superior recognition of emotions from body postures and vocal prosody. Consistent with this prediction, clinical groups with mimicry impairments - including Parkinson’s disease and alexithymia - show recognition difficulties extending across modalities (Prenger & MacDonald, 2018; Suslow & Kersting, 2021), suggesting a disrupted multimodal system. Second, all three recognition tasks in the current studies used clearly visible expressions; incorporating perceptually challenging stimuli (e.g., low-intensity morphs, noise-masked faces) would provide more targeted tests of when mimicry quality supports emotion understanding - conditions under which embodied simulation may be most beneficial. Third, comparing deliberate and spontaneous mimicry quality within the same individuals would clarify whether these two forms draw on shared representational resources, and how far findings generalize to the automatic mimicry most commonly linked to embodied simulation. Finally, given that correlations between mimicry lag and accuracy varied across emotions in our exploratory analyses - positive for sadness, negative for happiness - future research with greater emotion-specific power could clarify whether mimicry timing operates differently depending on the emotion expressed.

Taken together, the present results suggest a positive and replicable link between deliberate facial mimicry abilities and emotion-recognition efficiency. Both the accuracy and timing of mimicry independently contributed to recognition performance. By isolating mimicry as its own construct and relating it to both accuracy and drift-rate measures, this work helps clarify the role of embodied simulation in emotion perception and provides a framework for future clinical and basic research on individual differences in facial mimicry.

## Electronic Supplementary Material

Below is the link to the electronic supplementary material.

Supplementary Material 1

## Additional Information

### Data Availability

To protect participant privacy, we do not share the original facial mimicry video recordings. Instead, we provide the mimicry algorithm outputs, participant task performance data, and drift-diffusion model objects at “results_and_analysis_with_r/data” in: (https://osf.io/3rc92/files/sqk7z).

### Code Availability

Code for the analyses is available at: https://osf.io/3rc92/files/sqk7z.
