# Supplementary material for: Deliberate Facial Mimicry As a Skill That Predicts Emotion Recognition Performance
Source: Affect Sci. 2026 Apr 16;7(2):333–51. doi: 10.1007/s42761-026-00366-9 (PMC13269607; doi:10.1007/s42761-026-00366-9)
Supplement: Supplementary file 1 — Supplementary Material 1 [file 42761_2026_366_MOESM1_ESM.pdf]

Supplementary materials for "Deliberate Facial Mimicry As a Skill That Predicts Emotion Recognition Performance"

WTLCC Parameters:

We set the window size at 3 seconds with a maximum lag of 1 second for our WTLCC analysis. The 3-second window was chosen because it is sufficient to capture the essence of individual facial expressions, each of which lasted 4-5 seconds in the video stimuli. This window size was particularly critical given our experimental design: in at least one case, an actor's emotional expression (happiness) occurred at the end of a video clip, meaning both the stimulus and participant's recorded response required a window of 3 seconds combined with a maximum lag of 1 second to fully capture that expression. The 1-second maximum lag reflects the nature of deliberate mimicry, where participants were explicitly instructed to imitate the facial expressions as they were presented. Such intentional mimicry occurs relatively quickly (Korb et al., 2010; Hofree et al., 2014). While choosing WTLCC parameters is inherently challenging due to the multiple timescales at which relevant dynamics unfold (Moulder et al., 2018), our parameter selection was guided by the specific temporal structure of our stimuli and the deliberate nature of the mimicry task.

Algorithm Validation and Cutoff Determination:

Using all similarity scores from Study 1, we created two distributions: matched scores (correct actor–participant pairing) and null scores (pseudo-randomly mismatched pairings). The optimal cutpoint was determined by maximizing Youden's index (J = Sensitivity + Specificity − 1) across all possible thresholds. This approach balances false inclusions and exclusions and identifies where the matched and mismatched score distributions intersect (Youden, 1950; Fluss et al., 2005). We estimated this cutpoint non-parametrically and obtained a 95% bootstrap confidence interval by resampling scores with replacement 1,000 times (Schisterman et al., 2007). Using this cutoff, we filtered out all emotion × actor mimicry accuracy and mimicry lag values for each participant if the mimicry accuracy value was below the threshold.

The cutpoint analysis revealed strong discriminability between matched and null similarity distributions. The optimal threshold was 0.256 (95% CI: [0.231, 0.350]), yielding a Youden's index of J = 0.676. At this cutoff, the algorithm achieved a sensitivity of 87.1% for detecting true mimicry instances while maintaining a false-positive rate of 19.6% (See Supplemental Fig 1).

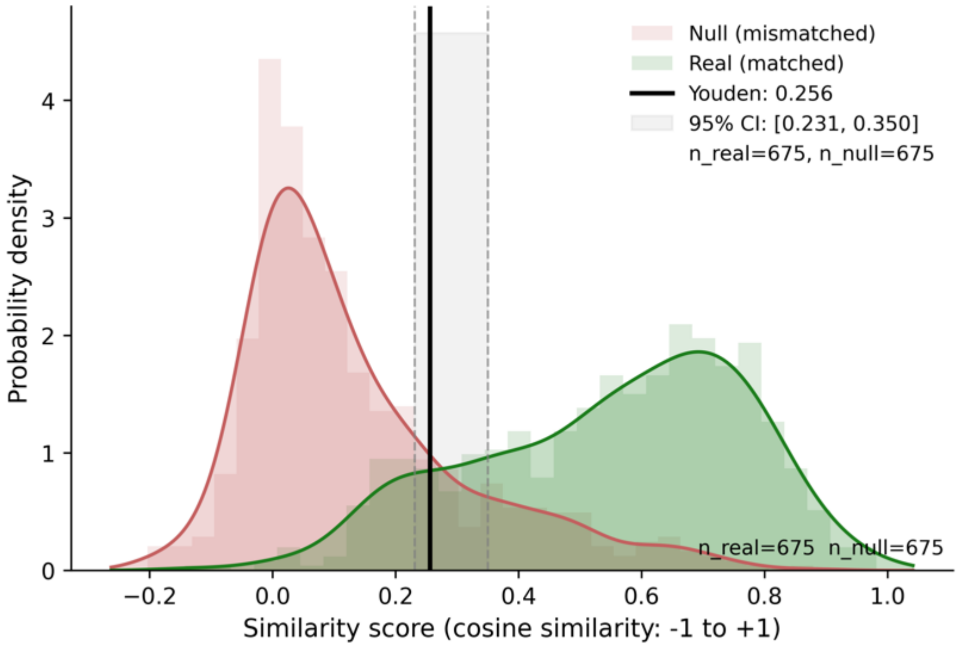

Supplemental Figure 1. Establishing a validity threshold for mimicry measurements. Kernel density estimates show the distribution of similarity scores for correctly matched actor–participant pairs (Real, green) and intentionally mismatched pairs (Null, red). The vertical black line marks the Youden-optimal cutpoint that maximizes sensitivity and specificity (J = sensitivity + specificity − 1), with the gray shaded region indicating the 95% nonparametric bootstrap confidence interval. The distributional separation illustrates how the threshold balances false inclusions and exclusions when identifying valid mimicry instances. Plot created using Python Matplotlib (Hunter, 2007).

Study 1 Drift-Diffusion Model Specifications and Convergence Diagnostics:

Models were fit in brms with the Wiener family; link functions were identity for drift-rate (v), log for boundary separation (a) and non-decision time (Ter), and logit for starting bias (w). Drift-rate centers were set from each task's mean accuracy using the approximation v ≈ logit(p)/(2a). Convergence was assessed via R-hat (target < 1.01) and effective sample sizes (ESS; reported as Bulk ESS / Tail ESS).

<u>Congruency Task</u> (N = 6,737 trials, 34 participants): drift-rate (v) prior = Student-t(df=3, center=0.438, scale=0.45). Boundary separation (a): Normal prior on the log scale centered to give median a ≈ 2.00 (SD on log scale ≈ 0.25). Non-decision time (Ter): Normal prior on the log scale centered at 0.70 s (SD ≈ 0.20), decomposed as 0.40 s enforced response lockout + 0.30 s sensory-motor overhead. Starting bias (w): Normal prior on the logit scale centered at 0.50 (SD ≈ 0.30). Group-level SDs on v for participant and emotion used scale ≈ 0.5. Convergence: All R-hat = 1.00. ESS: drift Intercept (685 / 1,253), bs Intercept (4,988 / 4,444), ndt Intercept (4,001 / 4,302), bias Intercept (4,663 / 3,931), participant SD (804 / 1,431), emotion SD (1,515 / 2,318).

<u>Stop-Motion Task</u> (N = 2,214 trials, 34 participants): drift-rate (v) prior = Student-t(df=3, center=0.395, scale=0.40). Boundary separation (a): Normal prior on the log scale with median a ≈ 2.00 (SD ≈ 0.30). Non-decision time (Ter): Normal prior on the log scale centered at 1.40 s (SD ≈ 0.20). Starting bias (w): Normal prior on the logit scale centered at 0.50 (SD ≈ 0.35). Group-level SDs (participant and emotion) used scale ≈ 0.6. Convergence: All R-hat = 1.00. ESS:

drift Intercept (1,806 / 2,895), bs Intercept (9,808 / 5,908), ndt Intercept (8,664 / 5,773), bias Intercept (10,568 / 6,205), participant SD (2,423 / 4,365), emotion SD (2,464 / 4,180).

Film Task (N = 1,972 trials, 34 participants): drift-rate (v) prior = Student-t(df=3, center=0.431, scale=0.40). Boundary separation (a): Normal prior on the log scale with median a ≈ 1.80 (SD ≈ 0.25). Non-decision time (Ter): Normal prior on the log scale centered on the leading edge of the RT distribution, defined as the 10th-percentile RT minus 0.20 s, constrained to 0.30–0.80 s (SD ≈ 0.20). Starting bias (w): Normal prior on the logit scale centered at 0.50 (SD ≈ 0.30). Participant-level SD on v used scale ≈ 0.6. Convergence: All R-hat = 1.00. ESS: drift Intercept (2,145 / 3,319), bs Intercept (9,140 / 6,048), ndt Intercept (8,873 / 6,307), bias Intercept (9,204 / 5,861), participant SD (2,576 / 4,126).

Study 2 Drift-Diffusion Model Specifications and Convergence Diagnostics (Informed Priors)

Models used the same links as Study 1 (identity for v, log for a and Ter, logit for w). Priors were informed by Study 1 posteriors (sequential Bayesian updating). Convergence was assessed via R-hat (target < 1.01) and effective sample sizes (ESS; reported as Bulk ESS / Tail ESS).

Congruency Task (N = 10,311 trials, 50 participants): drift-rate (v) prior = Student-t(df = 3, center = 1.25, scale = 0.45). Boundary separation (a): Normal prior on the log scale centered to give median a ≈ 1.77 (SD on log scale ≈ 0.25). Non-decision time (Ter): Normal prior on the log scale centered at 0.35 s (SD ≈ 0.20). Starting bias (w): Normal prior on the logit scale centered at 0.46 (SD ≈ 0.50). Group-level SDs on v: participant scale ≈ 0.70; emotion scale ≈ 2.50. *Convergence:* All R-hat = 1.00. ESS: drift Intercept (1,042 / 2,011), bs Intercept (7,091 / 5,844), ndt Intercept (5,939 / 5,947), bias Intercept (6,339 / 5,405), participant SD (915 / 2,052), emotion SD (1,569 / 2,624).

Stop-Motion Task (N = 3,210 trials, 50 participants): drift-rate (v) prior = Student-t(df = 3, center = 0.75, scale = 0.40). Boundary separation (a): Normal prior on the log scale centered to give median a ≈ 4.35 (SD ≈ 0.35). Non-decision time (Ter): Normal prior on the log scale centered at 0.63 s (SD ≈ 0.20). Starting bias (w): Normal prior on the logit scale centered at 0.34 (SD ≈ 0.50). Group-level SDs on v: participant scale ≈ 0.30; emotion scale ≈ 0.70. *Convergence:* All R-hat = 1.00. ESS: drift Intercept (1,847 / 2,953), bs Intercept (8,581 / 6,574), ndt Intercept (8,883 / 6,101), bias Intercept (8,397 / 5,700), participant SD (2,640 / 4,438), emotion SD (2,257 / 3,628).

Film Task (N = 3,050 trials, 50 participants): drift-rate (v) prior = Student-t(df = 3, center = 0.80, scale = 0.45). Boundary separation (a): Normal prior on the log scale centered to give median a ≈ 2.27 (SD ≈ 0.25). Non-decision time (Ter): Normal prior on the log scale centered at 0.92 s (SD ≈ 0.18). Starting bias (w): Normal prior on the logit scale centered at 0.50 (SD ≈ 0.50). Group-level SD on v (participant): scale ≈ 0.50. *Convergence:* All R-hat = 1.00. ESS: drift Intercept (3,846 / 5,218), bs Intercept (10,961 / 5,063), ndt Intercept (10,860 / 6,315), bias Intercept (11,976 / 6,228), participant SD (2,587 / 4,233).

Exploratory Combined-Sample Analysis: Associations Between Mimicry Metrics and DDM Decision Parameters - Model Specifications and Convergence Diagnostics

To examine relationships between mimicry metrics and DDM parameters beyond drift rate, we fitted hierarchical Bayesian drift-diffusion models to the combined dataset (N = 84 participants across both studies). Models used the same structure as the study-specific models: random intercepts for participant and (where applicable) emotion, with the Wiener likelihood and link functions of identity for drift rate, log for boundary separation and non-decision time, and logit for starting bias.

Priors for the combined models were informed by the Study 2 posteriors. Convergence was assessed via R-hat (all values < 1.01) and effective sample sizes (bulk ESS and tail ESS reported below).

<u>Congruency Task</u>*:* drift rate Intercept = 1.34 (SE = 0.21), R-hat = 1.00, Bulk ESS = 3408; boundary separation Intercept (log scale) = 0.46 (SE = 0.02), R-hat = 1.00, Bulk ESS = 1158; non-decision time Intercept (log scale) = -0.89 (SE = 0.02), R-hat = 1.00, Bulk ESS = 678.

<u>Stop-Motion Task</u>*:* drift rate Intercept = 0.79 (SE = 0.19), R-hat = 1.00, Bulk ESS = 2107; boundary separation Intercept (log scale) = 1.24 (SE = 0.02), R-hat = 1.00, Bulk ESS = 3149; non-decision time Intercept (log scale) = -1.27 (SE = 0.01), R-hat = 1.00, Bulk ESS = 9678.

<u>Film Task</u>*:* drift rate Intercept = 0.74 (SE = 0.03), R-hat = 1.00, Bulk ESS = 4116; boundary separation Intercept (log scale) = 0.67 (SE = 0.02), R-hat = 1.00, Bulk ESS = 1468; non-decision time Intercept (log scale) = 0.04 (SE = 0.02), R-hat = 1.01, Bulk ESS = 479.

Participant-level parameter estimates were extracted from each model and entered as outcomes in linear mixed-effects models predicting DDM parameters from mimicry metrics.

Reading the mind in the eye

A linear regression examined whether mimicry accuracy and mimicry lag predicted proportion correct in the Reading the Mind in the Eyes task; the overall model was not significant, F(2, 47) = 0.09, p = .916, R^2 = .004, with neither mimicry accuracy (b = -0.04, SE = 0.26, t = -0.14, p = .890) nor mimicry lag (b = -0.05, SE = 0.13, t = -0.42, p = .677) significantly predicting performance. Additionally, neither AQ scores (r = -.11, 95% CI [-.38, .17], p = .429) nor IRI scores (r = .15, 95% CI [-.13, .41], p = .301) were significantly correlated with RMET proportion correct. For RMET drift rates, a linear regression found no significant overall model, F(2, 47) = 1.09, p = .344, R^2 = .044, with neither mimicry accuracy (b = 0.54, SE = 0.53, t = 1.03, p = .309) nor mimicry lag (b = 0.34, SE = 0.27, t = 1.29, p = .202) significantly predicting drift rates. There were also no significant correlations between AQ scores and RMET drift rates (r = -.07, 95% CI [-.34, .21], p = .640) or between IRI scores and RMET drift rates (r = -.12, 95% CI [-.39, .16], p = .393).

A linear regression examined whether mimicry accuracy, mimicry lag, AQ total, and IRI total predicted confidence ratings (1-9 scale) in the Reading the Mind in the Eyes task. The overall model approached significance, F(4, 45) = 2.34, p = .070, R^2 = .17. Mimicry accuracy significantly predicted confidence ratings (b = 6.50, SE = 2.46, t = 2.64, p = .011, Cohen's f^2 = 0.17), indicating that higher mimicry accuracy was associated with greater confidence. However, mimicry lag (b = -0.23, SE = 1.29, t = -0.18, p = .858, Cohen's f^2 < 0.01), AQ total (b = -

0.02, SE = 0.03, t = -0.73, p = .468, Cohen's f^2 = 0.03), and IRI total (b = 0.01, SE = 0.02, t = 0.64, p = .528, Cohen's f^2 < 0.01) did not significantly predict confidence ratings.

Interaction analyses of mimicry accuracy and lag

To test whether the effects of mimicry accuracy and mimicry lag on emotion recognition are independent or conditional on one another, we examined their interaction. Predictors were mean-centered prior to computing the interaction term to ensure main effects are interpretable as effects at the mean of the other predictor.

For recognition accuracy, the main effects remained stable with the interaction term included. In Study 1, mimicry accuracy (b = 0.37, p = .010) and lag (b = 0.15, p = .034) both significantly predicted recognition accuracy; the interaction was not significant (b = -1.41, p = .361). In Study 2, mimicry accuracy (b = 0.22, p = .039) and lag (b = 0.11, p = .048) again both predicted recognition accuracy, with no significant interaction (b = -0.38, p = .688).

For drift rate, mimicry accuracy remained a significant predictor in both Study 1 (b = 1.73, p = .015) and Study 2 (b = 1.12, p = .016), while mimicry lag showed no association in either study (Study 1: b = 0.07, p = .826; Study 2: b = 0.15, p = .514). The interactions were not significant (Study 1: b = -5.75, p = .457; Study 2: b = -2.41, p = .564).

These results indicate that mimicry accuracy and lag exert additive, independent effects on emotion recognition performance. The absence of significant interactions supports modeling these predictors additively, as in the main analyses.

Exploratory emotion-specific mimicry–recognition relationships

Our primary analyses averaged mimicry accuracy across all five emotions to estimate a general mimicry skill. To explore whether emotion-specific mimicry predicts recognition of those same emotions, we tested this in two steps: first for recognition accuracy (proportion correct), then for drift-rate efficiency. In each model, mimicry accuracy for a given emotion served as the predictor for the recognition metric of that same emotion. Given that each emotion yielded only four mimicry instances per participant (one per actor), emotion-specific estimates are noisier than our aggregated measure, and all findings should be interpreted accordingly.

Recognition Accuracy. We fitted separate mixed-effects models per emotion, each predicting recognition accuracy from emotion-specific mimicry accuracy, with random intercepts for participant and task: *pc ~ mimicry_corr* + (1|*participant*) + (1|*task*). Anger mimicry significantly predicted anger recognition accuracy: *b* = 0.23, *SE* = 0.11, *t* = 2.20, *p* = .031. All other emotions showed weak, non-significant associations: sadness (*b* = 0.07, *SE* = 0.07, *p* = .308), surprise (*b* = 0.03, *SE* = 0.07, *p* = .715), happiness (*b* = −0.007, *SE* = 0.02, *p* = .764), and disgust (*b* = 0.04, *SE* = 0.15, *p* = .781). We note that happiness accuracy was near ceiling, which likely attenuated any observable correlation for that emotion.

Drift Rate. To obtain participant-level emotion-specific drift rates, we fitted separate Bayesian drift-diffusion models per emotion (one model per emotion, across both tasks for all 84

participants), extracting participant random effects as emotion-specific drift rate estimates. The resulting participant × emotion drift rates were then entered as outcomes in separate mixed-effects models per emotion, each predicting drift rate from emotion-specific mimicry accuracy, with random intercepts for participant and task. Anger and happiness emerged as significant predictors: anger (b = 0.90, SE = 0.36, t = 2.48, p = .015) and happiness (b = 0.61, SE = 0.25, t = 2.41, p = .018). The remaining emotions showed weaker, non-significant associations: surprise (b = 0.48, SE = 0.40, p = .231), sadness (b = 0.16, SE = 0.21, p = .458), and disgust (b = −0.11, SE = 0.34, p = .754).

Together, these exploratory analyses suggest that emotion-specific mimicry–recognition relationships exist but are inconsistent across emotions and outcome measures. Anger emerged as the most reliable emotion-specific predictor, appearing significantly in both accuracy and drift-rate analyses. Happiness showed an association with drift rate but not accuracy, likely reflecting ceiling effects in the proportion-correct measure. The overall pattern warrants caution given the low reliability of emotion-specific mimicry estimates (four instances per emotion per participant) and the exploratory nature of these analyses.

Supplemental Tables:

Supplemental Table 1: Study 1 Task performance

In the first three columns are the correlation between task performances (accuracy) in study 1, followed by mean and rt data on accuracy and RT per task.

Task	Congruency	Stop-Motion	Film	Mean Accuracy	SD Accuracy	Mean RT	SD RT
**Congruency**	1	0.425*	0.470**	0.852	0.049	0.504	0.159
**Stop-Motion**	0.425*	1	0.242	0.83	0.065	3.32	0.457
**Film**	0.470**	0.242	1	0.825	0.064	0.922	0.354

Supplemental Table 2: Study 2 Task performance

In the first three columns are the correlation between task performances (accuracy) in study 2, followed by mean and rt data on accuracy and RT per task.

Task	Congruency	Stop-Motion	Film	RMET	Mean Accuracy	SD Accuracy	Mean RT	SD RT
**Congruency**	1	0.427**	0.236	0.334*	0.847	0.056	0.458	0.131
**Stop-Motion**	0.427**	1	0.127	0.233	0.819	0.073	3.257	0.446
**Film**	0.236	0.127	1	0.403**	0.814	0.058	0.822	0.279
**RMET**	0.334*	0.233	0.403**	1	0.713	0.104	9.094	4.151

Supplemental Table 3: Study 1 mimicry quality and lag comparison

Mimicry quality (MQ) and mimicry lag (lag) correlations, mean and SD statistics.

Emotion	Correlation	Correlation p-value	n	Mean MQ	SD MQ	Mean Lag	SD Lag
**Overall (Average)**	-0.041	0.8188	34	0.602	0.052	0.653	0.107
**Anger**	-0.005	0.9574	119	0.624	0.168	0.602	0.385
**Disgust**	0.159	0.0865	117	0.553	0.158	0.694	0.365
**Happiness**	-0.208*	0.0273	113	0.648	0.192	0.609	0.362
**Sadness**	0.192*	0.0392	116	0.604	0.161	0.534	0.377
**Surprise**	0.132	0.1483	122	0.602	0.155	0.803	0.25

Supplemental Table 4: Study 2 mimicry quality and lag comparison

Study 2 Mimicry quality (MQ) and mimicry lag (lag) correlations, mean and SD statistics. Notably, the relationship between mimicry quality and lag is clearer than in study 1, as in this study we introduced controlled actor videos with breaks (neutral facial expression) between each emotion.

Emotion	Correlation	p-value	n	Mean Corr	SD Corr	Mean Lag	SD Lag
**Overall (Average)**	-0.259	0.0689	50	0.575	0.062	0.657	0.123
**Anger**	-0.178*	0.0349	141	0.537	0.12	0.688	0.21
**Disgust**	0.125	0.103	171	0.473	0.136	0.632	0.372
**Happiness**	-0.142	0.0787	155	0.774	0.23	0.53	0.325
**Sadness**	0.334***	0.0001	135	0.516	0.172	0.691	0.316
**Surprise**	-0.210**	0.0047	179	0.584	0.162	0.744	0.2

Separate task analysis:

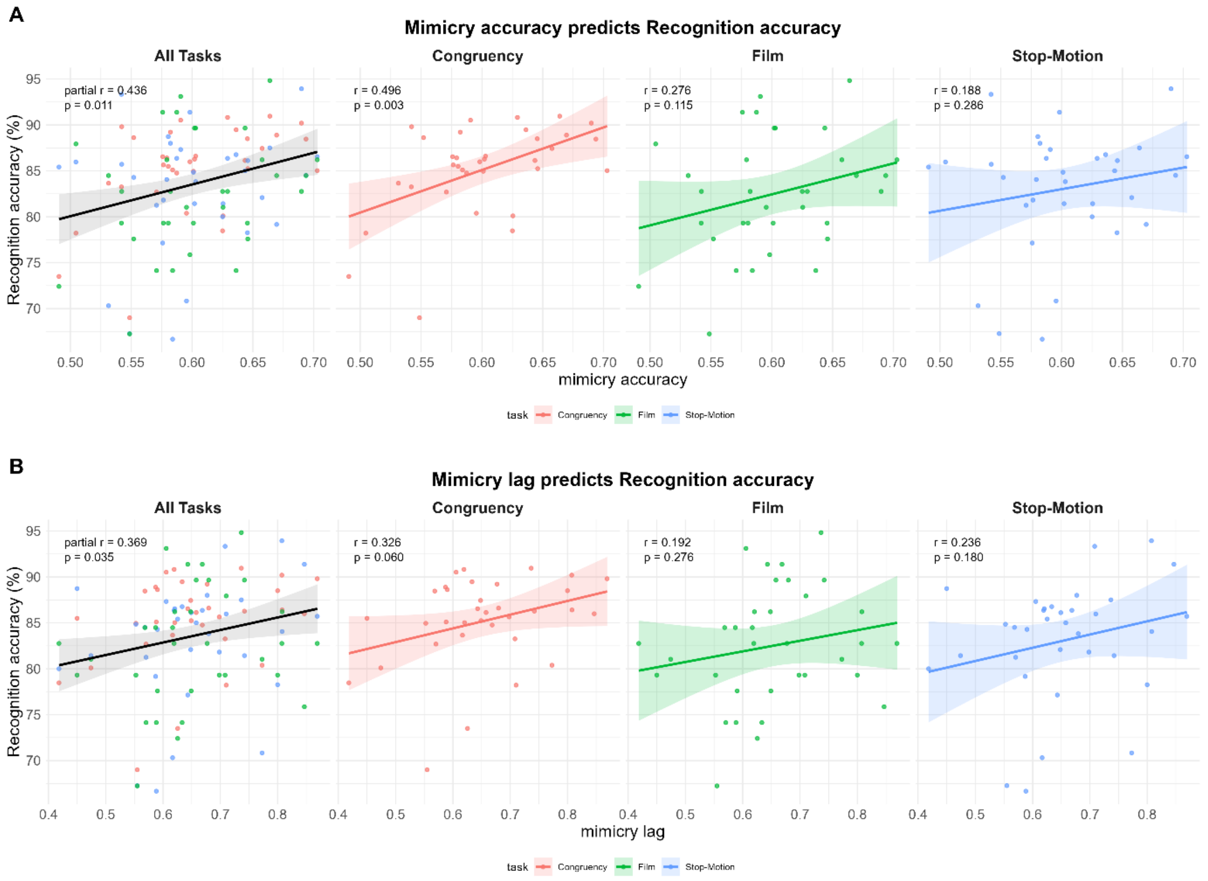

Supplemental Fig. 2 – Relationship between mimicry metrics and proportions correct (PC) by task in study 1. (A) Mimicry accuracy was positively associated with recognition accuracy across tasks. (B) Mimicry lag also showed a positive association with recognition accuracy. lines are least-squares fits with 95% CIs within facets. Text reports Pearson r within tasks and mixed-model partial r in the "All Tasks" panel. Figures were created with ggplot2 and assembled with cowplot (Wickham, 2016; Wilke, 2025).

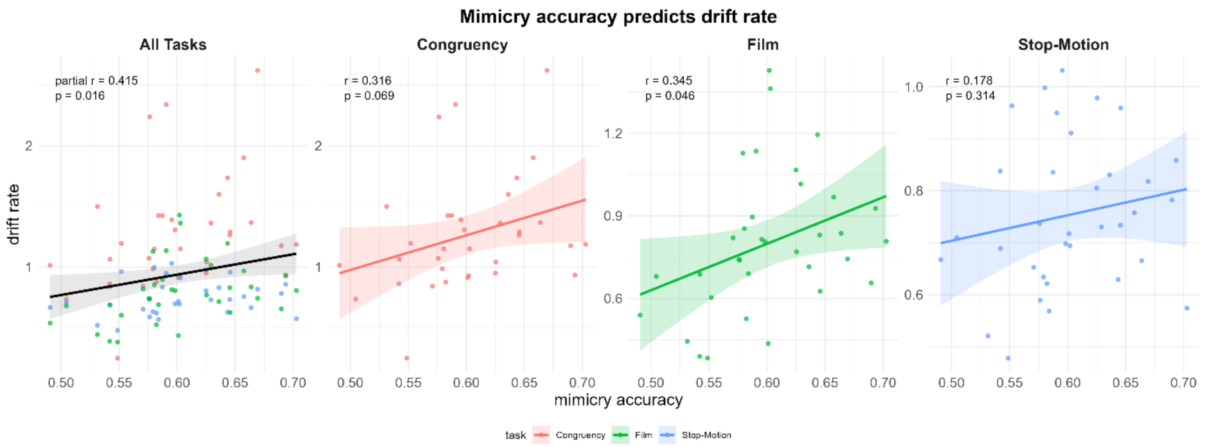

Supplemental Fig. 3 – Relationship between mimicry accuracy and drift-rate (v) by task in study 1. Mimicry accuracy is positively associated with drift rate across tasks. Lines are least-squares fits with 95% CIs within facets. The y-axis scale is free across panels to highlight within-task slopes (avoid vertical comparisons across facets). Text labels report Pearson r within tasks and mixed-model partial r in the "All Tasks" panel. Plot created with ggplot2 (Wickham, 2016).

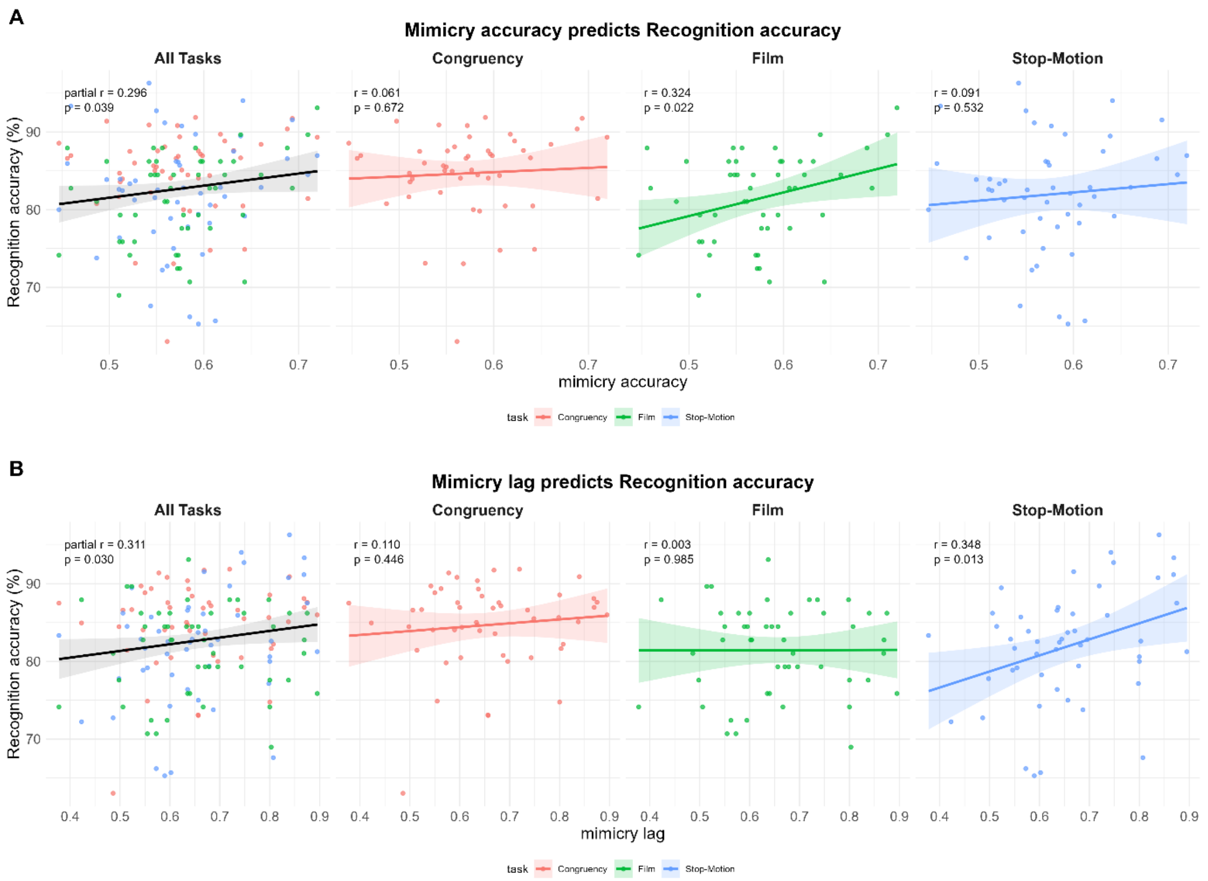

Supplemental Fig. 4 Relationship between mimicry metrics and proportions correct (PC) by task in study 2. (A) Mimicry accuracy was positively associated with recognition accuracy across tasks. (B) Mimicry lag also showed a positive association with recognition accuracy. lines are least-squares fits with 95% CIs within facets. Text reports Pearson r within tasks and mixed-model partial r in the "All Tasks" panel. Figures were created with ggplot2 and assembled with cowplot (Wickham, 2016; Wilke, 2025).

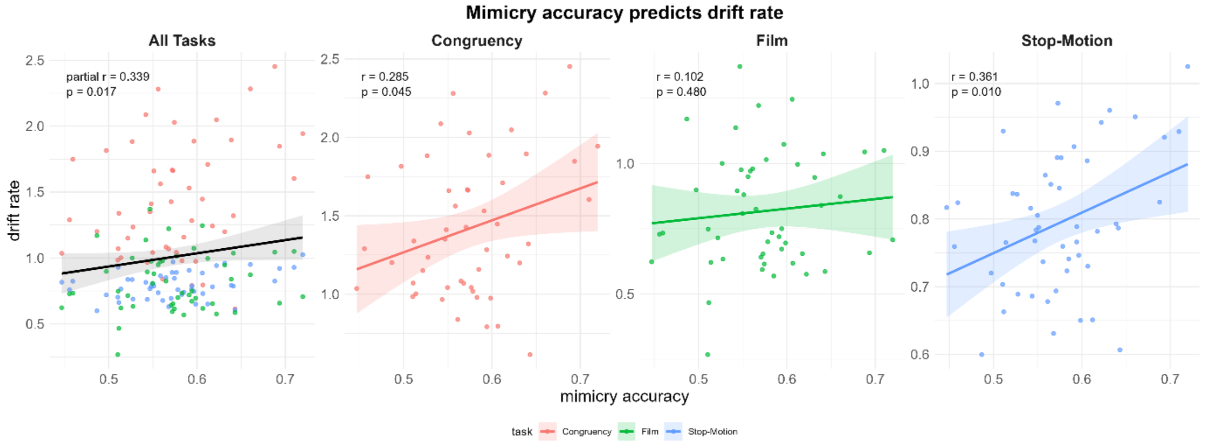

Supplemental Fig. 5 Relationship between mimicry accuracy and drift-rate (v) by task in study 2. Mimicry accuracy was positively associated with drift rate across tasks. Points show participant × task drift-rate estimates; lines are least-squares fits with 95% CIs within facets. The y-axis scale is free across panels to highlight within-task slopes (avoid vertical comparisons across facets). Text labels report Pearson r within tasks and mixed-model partial r in the "All Tasks" panel. Plot created with ggplot2 (Wickham, 2016).

Supplementary References:

Korb, S., Grandjean, D., & Scherer, K. R. (2010). Timing and voluntary suppression of facial mimicry to smiling faces in a Go/NoGo task - An EMG study. *Biological psychology*, *85*(2), 347-349.

Hofree, G., Ruvolo, P., Bartlett, M. S., & Winkielman, P. (2014). Bridging the mechanical and the human mind: spontaneous mimicry of a physically present android. *PloS one*, *9*(7), e99934.

Moulder, R. G., Boker, S. M., Ramseyer, F., & Tschacher, W. (2018). Determining synchrony between behavioral time series: An application of surrogate data generation for establishing falsifiable null-hypotheses. *Psychological methods*, *23*(4), 757.

Schisterman, E. F., & Perkins, N. (2007). Confidence intervals for the Youden index and corresponding optimal cut-point. *Communications in Statistics - Simulation and Computation®*, *36*(3), 549-563.
